# Systematic review of the use of process evaluations in knowledge translation research

**DOI:** 10.1186/s13643-019-1161-y

**Published:** 2019-11-07

**Authors:** Shannon D. Scott, Thomas Rotter, Rachel Flynn, Hannah M. Brooks, Tabatha Plesuk, Katherine H. Bannar-Martin, Thane Chambers, Lisa Hartling

**Affiliations:** 1 Faculty of Nursing, University of Alberta, Edmonton, Alberta Canada; 2 School of Nursing, Queen’s University, Kingston, Ontario Canada; 3 University of Alberta Libraries, Edmonton, Alberta Canada; 4 Department of Pediatrics, University of Alberta, Edmonton, Alberta Canada

**Keywords:** Process evaluation, Knowledge translation, Research use, Health interventions, KT interventions, Systematic review

## Abstract

**Background:**

Experimental designs for evaluating knowledge translation (KT) interventions can provide strong estimates of effectiveness but offer limited insight into *how* the intervention worked. Consequently, process evaluations have been used to explore the *causal mechanisms* at work; however, there are limited standards to guide this work. This study synthesizes current evidence of KT process evaluations to provide future methodological recommendations.

**Methods:**

Peer-reviewed search strategies were developed by a health research librarian. Studies had to be in English, published since 1996, and were not excluded based on design. Studies had to (1) be a process evaluation of a KT intervention study in primary health, (2) be a primary research study, and (3) include a licensed healthcare professional delivering or receiving the intervention. A two-step, two-person hybrid screening approach was used for study inclusion with inter-rater reliability ranging from 94 to 95%. Data on study design, data collection, theoretical influences, and approaches used to evaluate the KT intervention, analysis, and outcomes were extracted by two reviewers. Methodological quality was assessed with the Mixed Methods Appraisal Tool (MMAT).

**Results:**

Of the 20,968 articles screened, 226 studies fit our inclusion criteria. The majority of process evaluations used qualitative forms of data collection (43.4%) and individual interviews as the predominant data collection method. 72.1% of studies evaluated barriers and/or facilitators to implementation. 59.7% of process evaluations were stand-alone evaluations. The timing of data collection varied widely with post-intervention data collection being the most frequent (46.0%). Only 38.1% of the studies were informed by theory. Furthermore, 38.9% of studies had MMAT scores of 50 or less indicating poor methodological quality.

**Conclusions:**

There is widespread acceptance that the generalizability of quantitative trials of KT interventions would be significantly enhanced through *complementary* process evaluations. However, this systematic review found that process evaluations are of mixed quality and lack theoretical guidance. Most process evaluation data collection occurred post-intervention undermining the ability to evaluate the *process* of implementation. Strong science and methodological guidance is needed to underpin and guide the design and execution of process evaluations in KT science.

**Registration:**

This study is not registered with PROSPERO.

## Background

The implementation of research into healthcare practice is complex [1], with multiple levels to consider such as the patient, healthcare provider, multidisciplinary team, healthcare institution, and local and national healthcare systems. The implementation of evidence-based treatments to achieve healthcare system improvement that is robust, efficient, and sustainable is crucially important. However, it is well established that improving the availability of research is not enough for successful implementation [2]; rather, active knowledge translation (KT) interventions are essential to facilitate the implementation of research to practice. Determining the success of KT interventions and the implementation process itself relies on evaluation studies.

In the KT field, experimental designs such as randomized trials, cluster randomized trials, and stepped wedge designs are widely used for evaluating the effectiveness of KT interventions. Rigorous experimental designs can provide strong estimates of KT intervention effectiveness, but offer limited insight into how the intervention worked or not [1] as well as how KT interventions are mediated by different facilitators and barriers and how they lead to implementation or not [3–5]. KT interventions contain several interacting components, such as the degree of flexibility or tailoring of the intervention, the number of interacting components within the interventions, and the number and difficulty of behaviors required by those delivering or receiving the intervention [3]. This complexity makes it particularly challenging to evaluate KT intervention effectiveness [3–5]. The effectiveness of KT interventions is a result of the interactions between many factors such as context and mechanisms of change. A lack of intervention effect may be due to implementation failure rather than the ineffectiveness of the intervention itself. KT interventions pose methodological challenges and require augmentations to the standard experimental designs [6] to understand how they do or do not work.

As a result of these limitations, researchers have started to conduct process evaluations alongside experimental designs for evaluating KT interventions. The broad purpose of a process evaluation is to explore aspects of the implementation process [7]. Process evaluations can be used to assess the fidelity, dose, adaptation, reach, and quality of implementation [8, 9] and to identify the causal mechanisms [10, 11], mechanisms of impact [12], and contextual factors associated with variation in outcomes across sites [6, 13]. Furthermore, process evaluations can assist in interpreting the outcome results [7], the barriers and facilitators to implementation [14, 15] and sustainability [16], as well as examining the participants’ views [17] and understandings of components of the intervention [18, 19]. Process evaluations are vital in identifying the success or failure of implementation, which is critical in understanding intervention effectiveness.

Notwithstanding the work of Moore and colleagues [12], there have been scant methodological recommendations to guide KT process evaluations. This deficit has made designing process evaluations in KT research challenging and has hindered the potential for meaningful comparisons across process evaluation studies. In 2000, the Medical Research Council released an evaluation framework for designing and evaluating complex interventions; this report was later revised in 2008 [4, 20]. Of note, earlier guidance for evaluating complex interventions focused exclusively on randomized designs with no mention of process evaluations. The revisions mentioned process evaluations and the role that they can have with complex interventions, yet did not provide specific recommendations for evaluation designs, data collection types, time points, and standardized evaluation approaches for complex interventions. This level of specificity is imperative for research comparisons across KT intervention process evaluations and to understand how change is mediated by specific factors.

Recently, the Medical Research Council has commissioned an update of this guidance to be published in 2019 [21, 22]. The update re-emphasizes some of the previous messages related to complex intervention development and evaluation; however, it provides a more flexible and less linear model of the process with added emphasis to development, implementation, and evaluation phases as well as providing a variety of successful case examples that employ a range of methods (from natural experiments to clinical trials). Early reports of the update to the MRC framework highlight the importance of process and economic evaluations as good investments and a move away from experimental methods as the only or best option for evaluation.

In 2013, a framework for process evaluations for cluster-randomized trials of complex interventions was proposed by Grant and colleagues [20]; however, these recommendations were not based upon a comprehensive, systematic review of all approaches used by others. One study found that only 30% of the randomized controlled trails had associated qualitative investigations [23]. Moreover, a large proportion of those qualitative evaluations were completed before the trial, with smaller numbers of qualitative evaluations completed during the trial or following it. Given the limitations of the process evaluation work to date, it is critical to systematically review all existing process evaluations of KT outcome assessment. Doing so will aid in the development of rigorous methodological guidance for process evaluation research of KT interventions moving forward.

The aim of our systematic review is to synthesize the existing evidence on process evaluation studies assessing KT interventions. The purpose of our review is to make explicit the current state of methodological guidance for process evaluation research with the aim of providing recommendations for multiple end-user groups. This knowledge is critically important for healthcare providers, health quality consultants, decision and policy makers, non-governmental organizations, governmental departments, and health services researchers to evaluate the effectiveness of their KT efforts in order to ensure scarce healthcare resources are effectively utilized and enhanced knowledge is properly generalized to benefit others.

## Objectives and key questions

As per our study protocol [24] available openly via 10.1186/2046-4053-3-149, the objectives for this systematic review were to (1) systematically locate, assess, and report on published studies in healthcare that are a stand-alone process evaluation of a KT intervention or have a process evaluation component, and (2) offer guidance for researchers in terms of the development and design of process evaluations of KT interventions. The key research question guiding this systematic review was: what is the “state-of-the-science” of separate (stand-alone) or integrated process evaluations conducted alongside KT intervention studies?

## Methods

### Search strategy

This systematic review followed a comprehensive methodology using rigorous guidelines to synthesize diverse forms of research evidence [25], as outlined in our published protocol [24]. A peer-reviewed literature search was conducted by a health research librarian of English language articles published between 1996 and 2018 in six databases (Ovid MEDLINE/Ovid MEDLINE (R) In-Process & Other Non-Indexed Citations, Ovid EMBASE, Ovid PsycINFO, EBSCOhost CINAHL, ISI Web of Science, and ProQuest Dissertations and Theses). Full search details can be found in Additional file 1. See Additional file 2 for the completed PRISMA checklist.

### Inclusion/exclusion criteria

Studies were not excluded based upon research design and had to comply with three inclusion criteria (Table 1). A two-person hybrid approach was used for screening article titles and abstracts with inter-rater reliability ranging from 94 to 95%. Full-text articles were independently screened by two reviewers, and a two-person hybrid approach was used for data extraction.

**Table 1:** Process evaluation systematic review inclusion criteria

Study design	Must be a primary research study. Research studies including all designs, e.g., experimental, quasi-experimental, and non-experimental designs (e.g., case study). Opinion pieces, commentaries, methodological papers, book chapters, books, dissertations, conference abstracts, protocols, and reviews will not be included.
Study criteria	The study *is* or *includes a process evaluation* of a *health* implementation study/project that has a primary purpose of translating research into action/practice.^1^ The health (research) information disseminated must therefore be evidence-based. Studies must have clearly defined *knowledge translation strategies or interventions* to implement the health innovation. A *registered/licensed healthcare professional* or *allied healthcare professional* in medicine (physician, dentist), nursing, rehabilitation medicine (physiotherapy, occupational therapy, speech-language pathology), dietetics, or pharmacy must either *deliver or receive the intervention* (sensu Scott et al. 2011). A trainee healthcare professional (not yet licensed/registered) either delivering or receiving the intervention will be excluded if: a. The intervention is mandatory curricula for finishing their degree/gaining licensing. b. The intervention has no licensed healthcare professional involved. Process evaluations may be separate (*stand-alone)* or integrated (*embedded)* and must evaluate the knowledge translation strategies or interventions used to implement the evidence-based innovation (the process of implementation).
Outcome(s)	The process evaluation must be distinct from the primary outcomes of the KT/research implementation component. Where the paper is only reporting the process evaluation, this will be considered a distinct outcome.

^1^Health is defined according to the WHO (1946) conceptualization of a state of complete physical and mental well-being and not merely the absence of disease or infirmity, including prevention components and mental health but not “social health”

### Quality assessment

The methodological quality of all included studies was assessed using the Mixed Methods Appraisal Tool (MMAT) [26, 27] for quantitative, qualitative, and mixed methods research designs. The tool results in a methodological rating of 0, 25, 50, 75, and 100 (with 100 being the highest quality) for each study based on the evaluation of study selection bias, study design, data collection methods, sample size, intervention integrity, and analysis. We adapted the MMAT for multi-method studies (studies where more than one research approach was utilized, but the data were not integrated) by assessing the methods in the study individually and then choosing the lowest quality rating assigned. For studies where the process evaluation was integrated into the study design, the quality of the entire study was assessed.

### Data extraction, analysis, and synthesis

Study data were extracted using standardized Excel forms. Only data reported in included studies were extracted. Variables extracted included the following: (1) study design, (2) process evaluation type (integrated vs. separate), (3) process evaluation terms used, (4) timing of data collection (e.g., pre- and post-implementation of intervention), (5) KT intervention type, (6) KT intervention recipient, (7) target behavior, and (8) theory. Studies were grouped and synthesized according to each of the above variables. Evidence tables were created to summarize and describe the studies included in this review.

### Theoretical guidance

We extracted and analyzed data on any theoretical guidance that was identified and discussed for the process evaluation stage of the included studies. For the purpose of our systematic review, included studies were stated to be theoretically informed if the process evaluation used theory to (a) assist in the identification of appropriate outcomes, measures, and variables; (b) guide the evaluation of the KT process; and (c) identify potential predictors or mediators, or (d) as a framework for data analysis.

## Results

### Study design

**Fig. 1:**
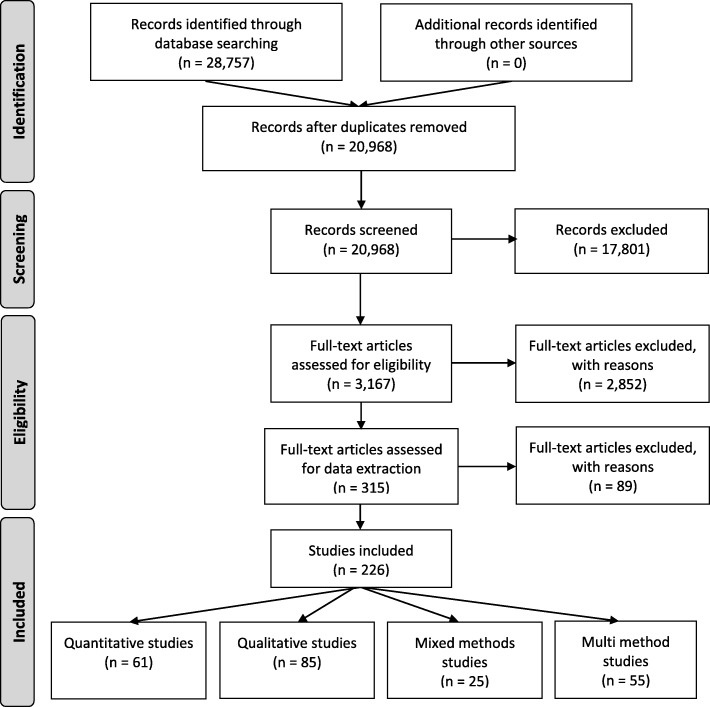
PRISMA flow diagram (Adapted from Moher et al. 2009)

Of the 20,968 articles screened, 226 full-text articles were included in our review (Fig. 1). See Additional file 3 for a full citation list of included studies.

Among these included articles, the following research designs were used: qualitative (*n* = 85, 37.6%), multi-methods (*n* = 55, 24.3%), quantitative descriptive (*n* = 44, 19.5%), mixed methods (*n* = 25, 11.1%), quantitative RCT (*n* = 14, 6.2%), and quantitative non-randomized (*n* = 3, 1.3%). See Table 2.

**Table 2:** Types of research design and associated quality of included studies (*n* = 226)

Study design	Number of studies (%)	MMAT score distribution				
		0	25	50	75	100
Mixed methods	25 (11.1)	2	1	8	11	3
Multi-methods	55 (24.3)	5	15	21	10	4
Qualitative	85 (37.6)	–	1	11	42	31
Quantitative descriptive	44 (19.5)	–	8	11	14	11
Quantitative non-randomized	3 (1.3)	–	1	–	1	1
Quantitative RCT	14 (6.2)	–	3	–	4	7

*RCT* randomized controlled trial

### Process evaluation type and terms

A total of 136 (60.2%) of the included studies were separate (stand-alone) process evaluations, while the process evaluations of the remaining studies (*n* = 90, 39.8%) were integrated into the KT intervention evaluation. Process evaluation research designs included the following: qualitative (*n* = 98, 43.4%), multi-methods (*n* = 56, 24.8%), quantitative descriptive (*n* = 51, 22.6%), and mixed methods (*n* = 21, 9.3%). See Table 3.

**Table 3:** Process evaluation research design of included studies (*n* = 226)

Process evaluation design	Number of studies (%)
Mixed methods	21 (9.3)
Multi-methods	56 (24.8)
Qualitative	98 (43.4)
Quantitative descriptive	51 (22.6)

The way in which each of the included studies described the purpose and focus of their process evaluation was synthesized and categorized thematically. Barriers and/or facilitators to implementation was the most widely reported term to describe the purpose and focus of the process evaluation (Table 4).

**Table 4:** Thematic analysis of process evaluation terms used in included studies (*n* = 226)

Process evaluation terms*	Number of studies
Acceptability	46
Adherence and fidelity	65
Attitudes	17
Barriers and facilitators	113
Barriers only	43
Contextual factors	25
Experiences and perceptions	87
Facilitators only	7
Feasibility	39
Feedback	16
Satisfaction	30
Sustainability and effectiveness	31

*Some studies used multiple terms to describe the process evaluation and its focus

### Methods and timing of data collection

Process evaluations had widespread variations in the methods of data collection, with individual interviews (*n* = 123) and surveys or questionnaires (*n* = 100) being the predominant methods (Table 5).

**Table 5:** Methods of data collection of included studies (*n* = 226)

Data collection methods*	Number of studies
Qualitative methods	
Individual interviews	123
Group interviews	15
Focus groups	51
Open-ended survey or questionnaires	14
Other	35
Quantitative methods	
Survey or questionnaire	100
Record Review	14
Other	37

*Some studies had more than one method of data collection

The majority of process evaluations collected data post-intervention (*n* = 104, 46.0%). The remaining studies collected data pre- and post-intervention (*n* = 40, 17.7%); during and post-intervention (*n* = 29, 12.8%); during intervention (*n* = 25, 11.1%); pre-, during, and post-intervention (*n* = 18, 7.9%); pre- and during intervention (*n* = 5, 2.2%); or pre-intervention (*n* = 3, 1.3%). In 2 studies (0.9%), the timing of data collection was unclear. See Table 6.

**Table 6:** Timing of data collection of included studies (*n* = 226)

Time of data collection	Number of studies (%)
Pre-intervention	3 (1.3)
Pre- and during intervention	5 (2.2)
Pre- and post-intervention	40 (17.7)
Pre-, during, and post-intervention	18 (7.9)
During and post-intervention	29 (12.8)
During intervention	25 (11.1)
Post-intervention	104 (46)
Unclear	2 (0.9)
Total	226 (100)

### Intervention details (type, recipient, and target behavior)

Most of the studies (*n* = 154, 68.1%) identified healthcare professionals (HCPs) as the exclusive KT intervention recipient, while the remaining studies had combined intervention recipients including HCP and others (*n* = 59, 26.1%), and HCP and patients (*n* = 13, 5.8%). Utilizing the Cochrane Effective Practice and Organisation of Care (EPOC) intervention classification schema [28], 218 (96.5%) studies had professional type interventions, 5 (2.2%) studies had professional type and organizational type interventions, and 3 (1.3%) studies had professional type and financial type interventions. The most common KT intervention target behaviors were “General management of a problem” (*n* = 132), “Clinical prevention services” (*n* = 45), “Patient outcome” (*n* = 35), “Procedures” (*n* = 33), and “Patient education/advice” (*n* = 32). See Table 7.

**Table 7:** Intervention details of included studies (*n* = 226)

KT intervention type	Number of studies (%)
Professional	218 (96.5)
Professional and organizational	5 (2.2)
Professional and financial	3 (1.3)
Total	226 (100)
KT intervention recipient type	
HCP	154 (68.1)
HCP and patients	13 (5.8)
HCP and others	59 (26.1)
Total	226 (100)
Target behavior of KT intervention*	
General management of a problem	132
Clinical prevention services	45
Patient outcome	35
Procedures	33
Patient education/advice	32
Prescribing	20
Test ordering	13
Diagnosis	11
Referrals	5
Record keeping	2
Professional-patient communication	1
Total	226

*Some studies had multiple targeted behaviors

### Theoretical guidance

Of the 226 studies, 38.1% (*n* = 86) were informed by theory (Table 8). The most frequently reported theories were as follows: (a) Roger’s Diffusion of Innovation Theory (*n* = 13), (b) Normalization Process Theory (*n* = 10), (c) Promoting Action on Research Implementation in Health Services Framework (*n* = 9), (d) Theory of Planned Behavior (*n* = 9), (e) Plan-Do-Study-Act Framework (*n* = 7), and (f) the Consolidated Framework for Implementation Research (*n* = 6).

**Table 8:** Theories used by theory-guided studies (*n* = 86)

Applied theories*	Number of studies
Roger’s theory/diffusion of innovation	13
Normalization process theory	10
Promoting Action on Research Implementation in Health Services framework	9
Theory of planned behavior	9
Plan-Do-Study-Act Framework	7
Theoretical Domains Framework	6
Consolidated Framework for Implementation Research	5
Reach, Effectiveness, Adoption, Implementation, and Maintenance Framework	4
Behavior Change Theory	3
Carrol et al. Framework for Intervention Fidelity	3
Grol and Wensing Theoretical Framework	3
Hulsher et al. Process Evaluation Framework	3
Medical Research Council Framework	3
Braun and Clarke Thematic Analysis in Psychology	2
Kirkpatrick and Kirkpatrick Training Program Evaluation Model	2
Ottawa Model of Research Use	2
Precede/Proceed Implementation Model	2
Prochaska and DiClemente Stages of Change Model	2
Other	19
Total	86

*Some studies had multiple theories guiding the process evaluation

### Quality assessment

The distribution of MMAT scores varied with study design (Table 2). The lowest scoring study design was multi-method, with 74.5% (*n* = 41) of multi-method studies scoring 50 or lower. Overall, many of the studies (*n* = 88, 38.9%) had an MMAT score of 50 or lower, with 29 (12.8%) studies scoring 25 and 7 (3.1%) studies scoring 0. Eighty-one studies (35.8%) scored 75, and 57 studies (25.2%) scored 100 (high quality). See Table 9.

**Table 9:** Distribution of MMAT scores (0 = lowest and 100 = highest score)

MMAT score distribution	Number of studies (%)
0	7 (3.1)
25	29 (12.8)
50	52 (23.1)
75	81 (35.8)
100	57 (25.2)
Total	226 (100)

## Discussion

Our findings provided many insights into the current practices of KT researchers conducting integrated or separate process evaluations, the focus of these process evaluations, the data collection considerations, and the poor methodological quality and a lack of theoretical guidance informing these process evaluations.

The majority of included studies (60.2%) conducted a separate (stand-alone) rather than integrated process evaluation. As Moore and colleagues suggest, there are advantages and disadvantages of either (separated or integrated) approach [12]. Arguments for separate process evaluations focus on analyzing process data without knowledge of outcome analysis to prevent biasing interpretations of results. Arguments for integration include ensuring implementation data is integrated into outcome analysis and using the process evaluation to identify intermediate outcome data and causal processes while informing the integration of new measures into outcome data collection. Our findings highlight that there is no clear preference for separate or integrated process evaluations. The decision for separation or integration of the process evaluation should be carefully considered by study teams to ensure it is the best option for their study objectives.

Our findings draw attention to a wide variety of terms and foci used within process evaluations. We identified a lack of clear and consistent concepts for process evaluations and their multifaceted components, as well as an absence of standard recommendations on how process evaluations should be developed and conducted. This finding is supported by a literature overview on process evaluations in public health published by Linnan and Steckler in 2002 [29]. We would encourage researchers to employ terms that are utilized by other researchers to facilitate making meaningful comparisons across studies in the future and to be mindful of comprehensively including the key components of a process evaluation, context, implementation, and mechanisms of impact [12].

Our findings highlight two important aspects about process evaluation data collection in relation to timing and type of data collected. In terms of data collection timing, almost half of the investigators collected their process evaluation data post-intervention (46%) without any pre-intervention or during intervention data collection. Surprisingly, only 17.7% of the included studies collected data pre- and post-intervention, and only 18 studies collected data pre-, during, and post-intervention. Process evaluations can provide useful information about intervention delivery and if the interventions were delivered as planned (fidelity), the intervention dose, as well as useful information about intervention reach and how the context shaped the implementation process. Our findings suggest a current propensity to collect data after intervention delivery (as compared to before and/or during). It is unclear if our findings are the result of a lack of forethought to employ data collection pre- and during implementation, a lack of resources, or a reliance on data collection approaches post-intervention. This aside, based upon our findings, we recommend that KT researchers planning process evaluations consider data collection earlier in the implementation process to prevent challenges with retrospective data collection and to maximize the potential power of process evaluations. Consideration of key components of process evaluations (context, implementation, and mechanisms of impact) is critically important to prevent inference-observation confusion from an exclusive reliance on outcome evaluations [12]. An intervention can have positive outcomes even when an intervention was not delivered as intended, as other events or influences can be shaping a context [30]. Conversely, an intervention may have limited or no effects for a number of reasons that extend beyond the ineffectiveness of the intervention including a weak research design or improper implementation of the intervention [31]. Implicitly, the process evaluation framework by Moore and colleagues suggests that process evaluation data collection ideally needs to be collected before and throughout the implementation process in order to capture all aspects of implementation [12].

In terms of data collection type, just over half (54.4%) of the studies utilized qualitative interviews as one form of data collection. Reflecting on the key components of process evaluations (context, implementation, and mechanisms of impact), the frequency of qualitative data collection approaches is lower than anticipated. Qualitative approaches such as interviewing are ideal for uncovering rich and detailed aspects of the implementation context, nuanced participant perspectives on the implementation processes, and the potential mediators to implementation impact. When considering the key components of a process evaluation (context, implementation, and mechanisms of impact), by default, it is suggestive of multi-method work. Consequently, we urge researchers to consider integrating qualitative and quantitative data into their process evaluation study designs to richly capture various perspectives. In addition to individual interviews, surveys, participant observation, focus groups, and document analysis could be used.

A major finding from this systematic review is the lack of methodological rigor in many of the process evaluations. Almost 40% of the studies included in this review had a MMAT score of 50 or less, but the scores varied significantly in terms of study designs used by the investigators. Moreover, the frequency of low MMAT scores for multi-method and mixed method studies suggests a tendency for lower methodological quality which could point to the challenging nature of these research designs [32] or a lack of reporting guidelines.

Our findings identified a lack of theoretical guidance employed and reported in the included process evaluation studies. It is important to note the role of theory within evaluation is considered contentious by some [33, 34], yet conversely, there are increasing calls for the use of theory in the literature. While there is this tension between using or not using theory in evaluations, there are many reported advantages to theory-driven evaluations [29, 33, 34], yet more than 60% of the included studies were not informed by theory. Current research evidence suggests that using theory can help to design studies that increase KT and enable better interpretation and replication of findings of implementation studies [35]. In alignment with Moore and colleagues, we encourage researchers to consider utilizing theory when designing process evaluations. There is no shortage of KT theories available. Recently, Strifler and colleagues identified 159 KT theories, models, and frameworks in the literature [36]. In the words of Moore and colleagues who were citing the revised MRC guidance (2008), “an understanding of the causal assumptions underpinning the intervention and use of evaluation to understand how interventions work in practice are vital in building an evidence base that informs policy and practice” [9].

### Limitations

As with all reviews, there is the possibility of incomplete retrieval of identified research; however, this review entailed a comprehensive search of published literature and rigorous review methods. Limitations include the eligibility restrictions (only published studies in the English language were included, for example), and data collection did not extend beyond data reported in included studies.

## Conclusions

The current state of the quality of evidence base of process evaluations in KT is weak. Policy makers and funding organizations should call for theory-based multi or mixed method designs with a complimentary process evaluation component. Mixed method designs, with an integrated process evaluation component, would help to inform decision makers about effective process evaluation approaches, and research funding organizations could further promote theory-based designs to guide the development and conduct of implementation studies with a rigorous process evaluation component. Achieving this goal may require well-assembled implementation teams including clinical experts, as well as strong researchers with methodological expertise.

We recommend that future investigators employ rigorous theory-guided multi or mixed method approaches to evaluate the processes of implementation of KT interventions. Our findings highlighted that to date, qualitative study designs in the form of separate (stand-alone) process evaluations are the most frequently reported approaches. The predominant data collection method of using qualitative interviews helps to better understand process evaluations and to answer questions about why the implementation processes work or not, but does not provide an answer about the effectiveness of the implementation processes used. In light of the work of Moore and colleagues [12], we advocate that future process evaluation investigators should use both qualitative and quantitative methods (mixed methods) with an integrated process evaluation component to evaluate implementation processes in KT research.

We identified the timing of data collection as another methodological weakness in this systematic review. It remains unclear why almost half of the included process evaluation studies collected data only post-implementation. To provide high-certainty evidence for process evaluations, we advocate for the collection of pre-, during, and post-implementation measures and the use of statistical uncertainty measures (e.g., standard deviation, standard error, *p* values, and confidence intervals). This would allow a rigorous assessment of the implementation processes and sound recommendations supported by statistical measures. The timing of pre-evaluations also helps to address issues before implementation occurs. There is widespread acceptance that the generalizability of quantitative trials of KT interventions would be significantly enhanced through complementary process evaluations. Most data collection occurred post-intervention undermining the ability to evaluate the *process* of implementation.

Strong science and methodological guidance is needed to underpin and guide the design and execution of process evaluations in KT science. A theory-based approach to inform process evaluations of KT interventions would allow investigators to reach conclusions, not only about the processes by which interventions were implemented and the outcomes they have generated, but also about the reliability of the causal assumptions that link intervention processes and outcomes. Future research is needed that could provide state-of-the-art recommendations on how to design, conduct, and report rigorous process evaluations as part of a theory-based mixed methods evaluation of KT projects. Intervention theory should be used to inform the design of implementation studies to investigate the success or failure of the strategies used. This could lead to more generalizable findings to inform researchers and knowledge users about effective implementation strategies.

## Supplementary information

**Additional file 1.** Peer-reviewed Search Strategy. Systematic literature review search documenation.

**Additional file 2.** PRISMA Checklist. Completed PRISMA checklist.

**Additional file 3.** Citation List of Included Studies. Citation list for studies included in this review.

## Data Availability

The datasets used and/or analyzed during the current study are available from the corresponding author on reasonable request.

## Abbreviations

EPOC: Effective Practice and Organisation of Care
HCP: Healthcare professional
KT: Knowledge translation
MMAT: Mixed Methods Appraisal Tool
RCT: Randomized controlled trial
