# Supplementary material for: Systematic review of the use of process evaluations in knowledge translation research
Source: Syst Rev. 2019 Nov 7;8:266. doi: 10.1186/s13643-019-1161-y (PMC6836407; doi:10.1186/s13643-019-1161-y)
Supplement: Supplementary file 3 — Additional file 3. Citation List of Included Studies. Citation list for studies included in this review. [file 13643_2019_1161_MOESM3_ESM.docx]

**Additional File 3:** Citation List of Included Studies.

1. Abbott CA, Dremsa T, Stewart DW, Mark DD, Swift CC. Adoption of a ventilator‐associated pneumonia clinical practice guideline. Worldviews on Evidence‐Based Nursing. 2006;3(4):139-52.

2. Abrahamson K, DeCrane S, Mueller C, Davila HW, Arling G. Implementation of a Nursing Home Quality Improvement Project to Reduce Resident Pain A Qualitative Case Study. 2015. p. 261-8.

3. Adrianna M, Michel B, Bayard R, Beverley S, Pablo P, Kiran J. Diabetes care in a complex humanitarian emergency setting: a qualitative evaluation. BMC Health Services Research. 2017(1):1.

4. Allanson ER, Grobicki K, Pattinson RC, Dickinson JE. Attitudes towards the implementation of universal umbilical artery lactate analysis in a South African district hospital. 2016.

5. Allen C, Jeffery H. Implementation and evaluation of a neonatal educational program in rural Nepal. Journal of tropical pediatrics. 2006;52(3):218-22.

6. Allison R, Lecky DM, McNulty CAM, Town K, Folkard KA, Dunbar JK, et al. Exploring why a complex intervention piloted in general practices did not result in an increase in chlamydia screening and diagnosis: a qualitative evaluation using the fidelity of implementation model. BMC Family Practice. 2017;18(1).

7. Anaya HD, Bokhour B, Feld J, Golden JF, Asch SM, Knapp H. Implementation of Routine Rapid HIV Testing Within the U.S. Department of Veterans Affairs Healthcare System. Journal for Healthcare Quality: Promoting Excellence in Healthcare. 2012;34(5):7-14.

8. Anna P, Susan B, Jonathan B, Stephen B, Anam P, Sandra I, et al. Improving reliability of clinical care practices for ventilated patients in the context of a patient safety improvement initiative. Journal of Evaluation in Clinical Practice. 2011(1):180.

9. Ansbro ÉM, Gill MM, Reynolds J, Shelley KD, Strasser S, Sripipatana T, et al. Introduction of syphilis point-of-care tests, from pilot study to national programme implementation in Zambia: a qualitative study of healthcare workers’ perspectives on testing, training and quality assurance. PloS one. 2015;10(6):e0127728.

10. Armstrong N, Herbert G, Brewster L. Contextual barriers to implementation in primary care: an ethnographic study of a programme to improve chronic kidney disease care. 2016. p. 426-31.

11. Atkins S, Lewin S, Ringsberg KC, Thorson A. Provider experiences of the implementation of a new tuberculosis treatment programme: A qualitative study using the normalisation process model. 2011.

12. August EM, Hayek S, Casillas D, Wortley P, Collins CB, Jr. Evaluation of the Dissemination, Implementation, and Sustainability of the "Partnership for Health" Intervention. 2016. p. E14-E8.

13. Avery L, Charman SJ, Taylor L, Flynn D, Mosely K, Speight J, et al. Systematic development of a theory-informed multifaceted behavioural intervention to increase physical activity of adults with type 2 diabetes in routine primary care: Movement as Medicine for Type 2 Diabetes. 2016.

14. Bach E, Beissner K, Murtaugh C, Trachtenberg M, Reid MC. Implementing a Cognitive-Behavioral Pain Self-Management Program in Home Health Care, Part 2: Feasibility and Acceptability Cohort Study. 2013. p. 130-7.

15. Bailie RS, Si D, Togni SJ, Robinson GW, d'Abbs PHN. A multifaceted health-service intervention in remote aboriginal communities: 3-year follow-up of the impact on diabetes care. Medical Journal of Australia. 2004;181(4):195-200.

16. Bain N, McKie L. Stages of Change training for opportunistic smoking intervention by the primary health care team. Part II: qualitative evaluation of long-term impact on professionals' reported behaviour. Health Education Journal. 1998;57(2):150-9.

17. Barnard JG, Dempsey AF, Brewer SE, Pyrzanowski J, O'Leary ST, Mazzoni SE. Facilitators and barriers to the use of standing orders for vaccination in obstetrics and gynecology settings. American Journal of Obstetrics and Gynecology. 2017;216(1):69.e1-.e7.

18. Bayley MT, Hurdowar A, Richards CL, Korner-Bitensky N, Wood-Dauphinee S, Eng JJ, et al. Barriers to implementation of stroke rehabilitation evidence: findings from a multi-site pilot project. Disability and rehabilitation. 2012;34(19):1633-8.

19. Beissner KL, Reid MC, Bach E, Murtaugh CM, Trifilio M, Barrón Y, et al. Evidence-based protocols: Into the home healthcare setting. Home Healthcare Now. 2017;35(2):105-12.

20. Beitz JM, Van Rijswijk L. Development and validation of an online interactive, multimedia wound care algorithms program. Journal of Wound Ostomy & Continence Nursing. 2012;39(1):23-34.

21. Bekkers M-J, Simpson SA, Dunstan F, Hood K, Hare M, Evans J, et al. Enhancing the quality of antibiotic prescribing in primary care: qualitative evaluation of a blended learning intervention. BMC family practice. 2010;11(1):34.

22. Bergin RJ, Grogan SM, Penberthy S, Mileshkin LR, Krishnasamy M, Aranda SK, et al. Developing an evidence-based, nurse-led psychoeducational intervention with peer support in gynecologic oncology. Cancer Nursing. 2016;39(2):E19-E30.

23. Beune EJ, Haafkens JA, Bindels PJ. Barriers and enablers in the implementation of a provider-based intervention to stimulate culturally appropriate hypertension education. Patient education and counseling. 2011;82(1):74-80.

24. Blewer AL, Delfin G, Leary M, Gaieski DF, Abella BS. A structured educational intervention to improve targeted temperature management utilization after cardiac arrest. Journal of Critical Care. 2013;28(3):259-64.

25. Bokhour BG, Saifu H, Goetz MB, Fix GM, Burgess J, Fletcher MD, et al. The role of evidence and context for implementing a multimodal intervention to increase HIV testing. Implementation Science. 2015;10(1):1-12.

26. Bradley EH, Schlesinger M, Webster TR, Baker D, Inouye SK. Translating research into clinical practice: Making change happen. Journal of the American Geriatrics Society. 2004(11):1875.

27. Brady MC, Stott DJ, Norrie J, Chalmers C, St George B, Sweeney PM, et al. Developing and evaluating the implementation of a complex intervention: using mixed methods to inform the design of a randomised controlled trial of an oral healthcare intervention after stroke. Trials. 2011;12(1):168.

28. Brown CH, Medoff D, Fang LJ, Lucksted A, Goldberg RW, Kreyenbuhl J, et al. Factors influencing implementation of smoking cessation treatment within community mental health centers. Journal of Dual Diagnosis. 2015;11(2):145-50.

29. Buffum MD, Buccheri R, Trygstad L, Gerlock AA, Birmingham P, Dowling GA, et al. Behavioral management of auditory hallucinations: Implementation and evaluation of a 10-week course. Journal of psychosocial nursing and mental health services. 2009;47(9):32-40.

30. Buffum MD, Buccheri RK, Trygstad LN, Dowling GA. Disseminating an Evidence-Based Course to Teach Self-Management of Auditory Hallucinations. Journal of psychosocial nursing and mental health services. 2014(4):32.

31. Cahill NE, Murch L, Cook D, Heyland DK, Canadian Critical Care Trials G. Implementing a multifaceted tailored intervention to improve nutrition adequacy in critically ill patients: results of a multicenter feasibility study. 2014.

32. Charlene W, Nanci M, Cherie B, Denise B, Mark A S. The role of information technology in translating educational interventions into practice: an analysis using the PRECEDE/PROCEED model. Journal of the American Medical Informatics Association. 2011(6):827.

33. Christiansen A, Roberts K. Integrating health and social care assessment and care management: findings from a pilot project evaluation. Primary Health Care Research & Development (Sage Publications, Ltd). 2005;6(3):269-77.

34. Chung B, Mikesell L, Miklowitz D. Flexibility and Structure May Enhance Implementation of Family-Focused Therapy in Community Mental Health Settings. 2014. p. 787-91.

35. Clarke DJ, Godfrey M, Hawkins R, Sadler E, Harding G, Forster A, et al. Implementing a training intervention to support caregivers after stroke: a process evaluation examining the initiation and embedding of programme change. Implementation Science. 2013;8(1):1-15.

36. Conn LG, McKenzie M, Pearsall EA, McLeod RS. Successful implementation of an enhanced recovery after surgery programme for elective colorectal surgery: a process evaluation of champions' experiences. 2015.

37. Connell LA, McMahon NE, Tyson SF, Watkins CL, Eng JJ. Mechanisms of action of an implementation intervention in stroke rehabilitation: a qualitative interview study. BMC Health Services Research. 2016;16:1-10.

38. Cooney D, Moon H, Liu Y, Miller RT, Perzynski A, Watts B, et al. A pharmacist based intervention to improve the care of patients with CKD: a pragmatic, randomized, controlled trial. 2015.

39. Coronado GD, Schneider JL, Petrik A, Rivelli J, Taplin S, Green BB. Implementation successes and challenges in participating in a pragmatic study to improve colon cancer screening: perspectives of health center leaders. 2017. p. 557-66.

40. Coulthard K, Patel D, Brizzolara C, Morriss R, Watson S. A feasibility study of expert patient and community mental health team led bipolar psychoeducation groups: implementing an evidence based practice. 2013.

41. Creed TA, Frankel SA, German RE, Green KL, Jager-Hyman S, Taylor KP, et al. Implementation of transdiagnostic cognitive therapy in community behavioral health: The Beck Community Initiative. Journal Of Consulting And Clinical Psychology. 2016;84(12):1116-26.

42. Cretin S, Farley DO, Dolter KJ, Nicholas W. Evaluating an integrated approach to clinical quality improvement: clinical guidelines, quality measurement, and supportive system design. Medical Care. 2001;39(8):II-70-II-84.

43. Curran JA, Brehaut J, Patey AM, Osmond M, Stiell I, Grimshaw JM. Understanding the Canadian adult CT head rule trial: use of the theoretical domains framework for process evaluation. 2013.

44. Dale S, Levi C, Ward J, Grimshaw JM, Jammali-Blasi A, D'Este C, et al. Barriers and enablers to implementing clinical treatment protocols for fever, hyperglycaemia, and swallowing dysfunction in the quality in acute stroke care (QASC) project-a mixed methods study. Worldviews on Evidence-Based Nursing. 2015;12(1):41-50.

45. Damschoder LJ, Goodrich DE, Robinson CH, Fletcher CE, Lowery JC. A systematic exploration of differences in contextual factors related to implementing the MOVE! weight management program in VA: A mixed methods study. 2011.

46. D'Aprano A, Silburn S, Johnston V, Oberklaid F, Tayler C. Culturally Appropriate Training for Remote Australian Aboriginal Health Workers: Evaluation of an Early Child Development Training Intervention. 2015. p. 503-11.

47. Darker CD, Sweeney B, Keenan E, Whiston L, Anderson R, Barry J. Screening and Brief Interventions for Illicit Drug Use and Alcohol Use in Methadone Maintained Opiate-Dependent Patients: Results of a Pilot Cluster Randomized Controlled Trial Feasibility Study. Substance Use & Misuse. 2016;51(9):1104-15.

48. Darney BG, Weaver MR, VanDerhei D, Stevens NG, Prager SW. "One of those areas that people avoid" a qualitative study of implementation in miscarriage management. BMC Health Services Research. 2013;13(1):1-9.

49. De Vos MLG, Van Der Veer SN, Graafmans WC, De Keizer NF, Jager KJ, Westert GP, et al. Process evaluation of a tailored multifaceted feedback program to improve the quality of intensive care by using quality indicators. BMJ quality & safety (Print). 2013(3):233.

50. Deprez R, Kinner A, Millard P, Baggott L, Mellett J, Loo JL. Improving quality of care for patients with chronic obstructive pulmonary disease. Population health management. 2009;12(4):209-15.

51. Desveaux L, Saragosa M, Rogers J, Bevan L, Loshak H, Moser A, et al. Improving the appropriateness of antipsychotic prescribing in nursing homes: a mixed-methods process evaluation of an academic detailing intervention. Implementation Science. 2017(1):1.

52. Dopp CME, Graff MJL, Rikkert MGMO, van der Sanden MWGN, Vernooij-Dassen MJFJ. Determinants for the effectiveness of implementing an occupational therapy intervention in routine dementia care. 2013.

53. Draper CE, Nemutandani SM, Grimsrud AT, Rudolph M, Kolbe-Alexander TL, de Kock L, et al. Qualitative evaluation of a physical activity-based chronic disease prevention program in a low-income, rural South African setting. Rural & Remote Health. 2010;10(3):1-14.

54. Duff J, Omari A, Walker K, Middleton S, McInnes E. Educational outreach visits to improve venous thromboembolism prevention in hospitalised medical patients: A prospective before-and-after intervention study. BMC Health Services Research. 2013;13(1).

55. Dykes F, Richardson-Foster H, Crossland N, Thomson G. ‘Dancing on a thin line’: Evaluation of an infant feeding information team to implement the WHO code of marketing of breast-milk substitutes. Midwifery. 2012;28(6):765-71.

56. Dynes M, Rahman A, Beck D, Moran A, Rahman A, Pervin J, et al. Home-based life saving skills in Matlab, Bangladesh: a process evaluation of a community-based maternal child health programme. Midwifery. 2011;27(1):15-22.

57. Edvardsson K, Garvare R, Ivarsson A, Eurenius E, Mogren I, Nyström ME. Sustainable practice change: Professionals' experiences with a multisectoral child health promotion programme in Sweden. BMC Health Services Research. 2011;11(1):61-72.

58. Eldh AC, Tollne A, Förberg U, Wallin L. What Registered Nurses Do and Do Not in the Management of Pediatric Peripheral Venous Catheters and Guidelines: Unpacking the Outcomes of Computer Reminders. Worldviews on Evidence-Based Nursing. 2016;13(3):207-15.

59. El-Mallakh P, Howard PB, Bond GR, Roque AP. Challenges of implementing a medication management evidence-based practice in a community mental health setting: results of a qualitative study. Issues In Mental Health Nursing. 2014;35(7):517-25.

60. Eva S, Lennart F, Lena G, Anna‐Greta M. Getting evidence‐based pressure ulcer prevention into practice: a process evaluation of a multifaceted intervention in a hospital setting. Journal of Clinical Nursing. 2017(19-20):3200.

61. Evans RE, Price S. Exploring organisational influences on the implementation of gatekeeper training: A qualitative study of the Applied Suicide Intervention Skills Training (ASIST) programme in Wales. Critical public health. 2013;23(2):213-24.

62. Fairchild E, Roberts L, Zelman K, Michelli S, Hastings-Tolsma M. Implementation of Robert's Coping with Labor Algorithm© in a large tertiary care facility. Midwifery. 2017;50:208-18.

63. Feibelmann S, Yang TS, Uzogara EE, Sepucha K. What does it take to have sustained use of decision aids? A programme evaluation for the Breast Cancer Initiative. Health Expectations. 2011;14:85-95.

64. Finn Davis K, Napolitano N, Li S, Pinto M, Buffman H, Nadkarni V, et al. Promoters and barriers to implementation of tracheal intubation airway safety bundle: A mixed-method analysis. Pediatric Critical Care Medicine. 2017;18(10):965-72.

65. Fisher AR. Development of clinical practice guidelines for urinary continence care of adult stroke survivors in acute and rehabilitation settings. Canadian Journal of Neuroscience Nursing. 2014;36(3):16-31.

66. Flottorp S, Oxman AD. Identifying barriers and tailoring interventions to improve the management of urinary tract infections and sore throat: a pragmatic study using qualitative methods. BMC Health Services Research. 2003;3(1):3.

67. Forsner T, Hansson J, Brommels M, Wistedt AÅ, Forsell Y. Implementing clinical guidelines in psychiatry: a qualitative study of perceived facilitators and barriers. BMC Psychiatry. 2010;10:1-10.

68. Francis JJ, Eccles MP, Johnston M, Whitty P, Grimshaw JM, Kaner EF, et al. Explaining the effects of an intervention designed to promote evidence-based diabetes care: a theory-based process evaluation of a pragmatic cluster randomised controlled trial. Implementation Science. 2008;3(1):50.

69. Francis NA, Phillips R, Wood F, Hood K, Simpson S, Butler CC. Parents' and clinicians' views of an interactive booklet about respiratory tract infections in children: a qualitative process evaluation of the EQUIP randomised controlled trial. BMC Family Practice. 2013;14:182-.

70. Franx G, Oud M, de Lange J, Wensing M, Grol R. Implementing a stepped-care approach in primary care: results of a qualitative study. 2012.

71. French B, Thomas LH, Harrison J, Forshaw D, Watkins CL, Burton CR, et al. Implementing a Systematic Voiding Program for Patients with Urinary Incontinence after Stroke. Qualitative Health Research. 2016;26(10):1393-408.

72. Fretheim A, Håvelsrud K, Oxman AD. Rational Prescribing in Primary care (RaPP): process evaluation of an intervention to improve prescribing of antihypertensive and cholesterol-lowering drugs. Implementation Science. 2006;1:19-9.

73. Geoffrey DB, Sevan M, Scott K, Elizabeth AJ, Brian H, Eva K-R, et al. Barriers and facilitators to reducing frequent laboratory testing for patients who are stable on warfarin: a mixed methods study of de-implementation in five anticoagulation clinics. Implementation Science. 2017(1):1.

74. Georgeu D, Colvin CJ, Lewin S, Fairall L, Bachmann MO, Uebel K, et al. Implementing nurse-initiated and managed antiretroviral treatment (NIMART) in South Africa: a qualitative process evaluation of the STRETCH trial. Implementation Science. 2012;7(1):66.

75. Gerald LB, Bruce F, Brooks CM, Brook N, Kimerling ME, Windsor RA, et al. Standardizing contact investigation protocols. Tuberculosis contact investigations. 2003(12):S369.

76. Gerrish K, Laker S, Taylor C, Kennedy F, McDonnell A. Enhancing the quality of oral nutrition support for hospitalized patients: a mixed methods knowledge translation study (The EQONS study). 2016. p. 3182-94.

77. Gesthalter YB, Koppelman E, Bolton R, Slatore CG, Yoon SH, Cain HC, et al. Evaluations of Implementation at Early-Adopting Lung Cancer Screening Programs: Lessons Learned. Chest. 2017;152(1):70-80.

78. Gingold JA, Briccetti C, Zook K, Gillespie CW, Gubernick RS, Moon RY, et al. Context Matters: Practitioner Perspectives on Immunization Delivery Quality Improvement Efforts. 2016. p. 825-37.

79. Goodfellow J, Agarwal S, Harrad F, Shepherd D, Morris T, Ring A, et al. Cluster randomised trial of a tailored intervention to improve the management of overweight and obesity in primary care in England. 2016.

80. Graham ID, Logan J, Davies B, Nimrod C. Changing the use of electronic fetal monitoring and labor support: a case study of barriers and facilitators. Birth. 2004;31(4):293-301.

81. Gray AZ, Soukaloun D, Soumphonphakdy B. A qualitative study of provider perceptions of influences on uptake of pediatric hospital guidelines in Lao PDR. American Journal of Tropical Medicine and Hygiene. 2017;97(2):602-10.

82. Gray F, Spence W, Kelly D. Cultivation of a learning culture in general practice: an educational intervention. Education for Primary Care. 2010;21(5):290-8.

83. Grimshaw JM, Graham ID, Robinson N, Presseau J, Eccles MP, Tetroe J, et al. Looking inside the black box: Results of a theory-based process evaluation exploring the results of a randomized controlled trial of printed educational messages to increase primary care physicians' diabetic retinopathy referrals [Trial registration number ISRCTN72772651]. Implementation Science. 2014;9(1).

84. Guest EM, Keatinge DR, Reed J, Johnson KR, Higgins HM, Greig J. Implementing and evaluating a professional practice framework in child and family health nursing: A pilot project. Nurse Education in Practice. 2013;13(5):393-9.

85. Guo F-R, Hung L-Y, Chang C-J, Leung K-K, Chen C-Y. The evaluation of a Taiwanese training program in smoking cessation and the trainees' adherence to a practice guideline. BMC public health. 2010;10(1):77.

86. Hälleberg Nyman M, Forsman H, Wallin L, Ostaszkiewicz J, Hommel A, Eldh AC. Promoting evidence‐based urinary incontinence management in acute nursing and rehabilitation care—A process evaluation of an implementation intervention in the orthopaedic context. Journal of Evaluation in Clinical Practice. 2019;25(2):282-9.

87. Hammelef KJ, Friese CR, Breslin TM, Riba M, Schneider SM. Implementing Distress Management Guidelines in Ambulatory Oncology. Clinical Journal of Oncology Nursing. 2014;18:31-6.

88. Hammond A, Klompenhouwer P. Getting evidence into practice: implementing a behavioural joint protection education programme for people with rheumatoid arthritis. British Journal of Occupational Therapy. 2005;68(1):25-33.

89. Hanbury A, Wallace LM, Clark M. Multiple outcome measures and mixed methods for evaluating the effectiveness of theory-based behaviour-change interventions: A case study targeting health professionals' adoption of a national suicide prevention guideline. Psychology, health & medicine. 2011(3):291.

90. Hannon PA, Vu T, Yette E, Bowen DJ, Ogdon S, Fleury EM, et al. Implementation and Process Evaluation of a Workplace Colorectal Cancer Screening Program in Eastern Washington. Health Promotion Practice. 2013;14(2):220-7.

91. Harris FM, Maxwell M, O’Connor RC, Coyne J, Arensman E, Székely A, et al. Developing social capital in implementing a complex intervention: a process evaluation of the early implementation of a suicide prevention intervention in four European countries. BMC Public Health. 2013;13(1):158.

92. Hawkes C, Foxcroft DR, Yerrell P. Clinical guideline for nurse‐led early extubation after coronary artery bypass: an evaluation. Journal of advanced nursing. 2010;66(9):2038-49.

93. Helfrich CD, Savitz LA, Swiger KD, Weiner BJ. Adoption and implementation of mandated diabetes registries by community health centers. American journal of preventive medicine. 2007;33(1):S50-S65.

94. Henihan AM, McCombe G, Klimas J, Swan D, Leahy D, Anderson R, et al. Feasibility of alcohol screening among patients receiving opioid treatment in primary care. 2016.

95. Hermens R, Hak E, Hulscher M, Braspenning J, Grol R. Adherence to guidelines on cervical cancer screening in general practice: programme elements of successful implementation. Br J Gen Pract. 2001;51(472):897-903.

96. Hetlevik I, Holmen J, Kruger O, Kristensen P, Iversen H, Furuseth K. Implementing clinical guidelines in the treatment of diabetes mellitus in general practice - Evaluation of effort, process, and patient outcome related to implementation of a computer-based decision support system. 2000. p. 210-27.

97. Hooker L, Small R, Taft A, Humphreys C, Hegarty K. Applying normalization process theory to understand implementation of a family violence screening and care model in maternal and child health nursing practice: A mixed method process evaluation of a randomised controlled trial. Implementation Science. 2015;10(1).

98. Huis A, Holleman G, van Achterberg T, Grol R, Schoonhoven L, Hulscher M. Explaining the effects of two different strategies for promoting hand hygiene in hospital nurses: a process evaluation alongside a cluster randomised controlled trial. Implementation Science. 2013;8(1):41.

99. Huntink E, Wensing M, Timmers IM, van Lieshout J. Process evaluation of a tailored intervention programme of cardiovascular risk management in general practices. Implementation Science. 2016;11:1-11.

100. Jaiantilal P, Gutin SA, Cummings B, Mbofana F, Rose CD. Acceptability, feasibility and challenges of implementing an HIV prevention intervention for people living with HIV/AIDS among healthcare providers in Mozambique: Results of a qualitative study. Sahara J. 2015;12(1):2-9.

101. Jan A, Ellen H, Linda O-P. Implementing evidence-based nursing practice: a tale of two intrapartum nursing units. Nursing Inquiry. 2003(4):218.

102. Johnson MJ, Leaf AA, Pearson F, Clark HW, Dimitrov BD, Pope C, et al. Successfully implementing and embedding guidelines to improve the nutrition and growth of preterm infants in neonatal intensive care: A prospective interventional study. BMJ Open. 2017;7(12).

103. Jonathan B V. Process evaluation of an educational intervention to improve end-of-life care: The Education for Physicians on End-of-Life Care (EPEC) Program. American Journal of Hospice & Palliative Medicine. 2001(4):233.

104. Jones F, Livingstone E, Hawkes L. ‘Getting the Balance between Encouragement and Taking Over’—Reflections on Using a New Stroke Self‐Management Programme. Physiotherapy Research International. 2013;18(2):91-9.

105. Josephs SA, Lemmink GA, Strong JA, Barry CL, Hurford WE. Improving Adherence to Intraoperative Lung-Protective Ventilation Strategies at a University Medical Center. 2018. p. 150-60.

106. Judith G, Gina F, Karen A, Mary Ellen W. Process Evaluation of a Nurse-Delivered Smoking Relapse Prevention Program for New Mothers. Journal of Community Health Nursing. 2005;22(3):157.

107. Kaasalainen S, Ploeg J, Donald F, Coker E, Brazil K, Martin-Misener R, et al. Positioning Clinical Nurse Specialists and Nurse Practitioners as Change Champions to Implement a Pain Protocol in Long-Term Care. Pain Management Nursing. 2015;16(2):78-88.

108. Kapp S. Successful implementation of clinical practice guidelines for pressure risk management in a home nursing setting. 2013. p. 895-901.

109. Karen J, Alan Q, Sue P, Geoff B, Duncan S. Quality of intervention delivery in a cluster randomised controlled trial: a qualitative observational study with lessons for fidelity. Trials. 2017(1):1.

110. Katz DA, Holman J, Prochazka A, Battaglia C, Titler M, Vander Weg MW, et al. Implementing Smoking Cessation Guidelines for Hospitalized Veterans: Effects on Nurse Attitudes and Performance. Journal of general internal medicine. 2013(11):1420.

111. Katz DA, Paez MW, Reisinger HS, Gillette MT, Weg MW, Titler MG, et al. Implementation of smoking cessation guidelines in the emergency department: a qualitative study of staff perceptions. Addiction science & clinical practice. 2014;9:1.

112. Katz DA, Stewart K, Paez M, Holman J, Adams SL, Vander Weg MW, et al. "Let Me Get You a Nicotine Patch": Nurses' Perceptions of Implementing Smoking Cessation Guidelines for Hospitalized Veterans. 2016. p. 373-82.

113. Kavanagh T, Stevens B, Seers K, Sidani S, Watt-Watson J. Process evaluation of appreciative inquiry to translate pain management evidence into pediatric nursing practice. Implementation Science. 2010;5:90-102.

114. Kennedy A, Rogers A, Chew-Graham C, Blakeman T, Bowen R, Gardner C, et al. Implementation of a self-management support approach (WISE) across a health system: a process evaluation explaining what did and did not work for organisations, clinicians and patients. Implementation science : IS. 2014;9:129.

115. Kennedy CC, Ioannidis G, Adachi JD, Papaioannou A, Thabane L. Implementing a Knowledge Translation Intervention in Long-Term Care: Feasibility Results From the Vitamin D and Osteoporosis Study (ViDOS). Journal of the American Medical Directors Association. 2014;15(12):943-5.

116. Kerr S, Whyte R, Watson H, Tolson D, McFadyen AK. A Mixed-Methods Evaluation of the Effectiveness of Tailored Smoking Cessation Training for Healthcare Practitioners Who Work with Older People. Worldviews on Evidence-Based Nursing. 2011;8(3):177-86.

117. Khodyakov D, Ridgely MS, Huang C, DeBartolo KO, Sorbero ME, Schneider EC. Project JOINTS: What factors affect bundle adoption in a voluntary quality improvement campaign? BMJ Quality and Safety. 2015;24(1):38-47.

118. Kim B-H, Kim H-S, Yu S-J, Choi S-E, Jung Y, Kwon S-H. Evaluation of End-of-Life Nursing Education Consortium-Geriatric Train-the-Trainer Program in Korea. Korean Journal of Adult Nursing. 2012;24(4):390-7.

119. Kim YM, Park KN, Lee SJ, Jo SJ. Implementation of the guidelines for targeted temperature management after cardiac arrest: A longitudinal qualitative study of barriers and facilitators perceived by hospital resuscitation champions. BMJ Open. 2016;6(1).

120. Kwok YLA, Harris P, McLaws M-L. Social cohesion: The missing factor required for a successful hand hygiene program. AJIC: American Journal of Infection Control. 2017;45(3):222-7.

121. Lambeek LC, van Mechelen W, Buijs PC, Loisel P, Anema JR. An integrated care program to prevent work disability due to chronic low back pain: a process evaluation within a randomized controlled trial. 2009.

122. Lapointe NM, Kramer JM, Weinfurt KP, Califf RM. Practitioner acceptance of the dofetilide risk-management program. Pharmacotherapy. 2002(8):1041.

123. Lee HC, Arora V, Brown T, Lyndon A. Thematic analysis of barriers and facilitators to implementation of neonatal resuscitation guideline changes. 2017. p. 249-53.

124. Legris M-e, Seguin NC, Desforges K, Sauve P, Lord A, Bell R, et al. Pharmacist Web-Based Training Program on Medication Use in Chronic Kidney Disease Patients: Impact on Knowledge, Skills, and Satisfaction. Journal of Continuing Education in the Health Professions. 2011;31(3):140-50.

125. Lemmens KM, Nieboer AP, Rutten-Van Mölken MP, van Schayck CP, Asin JD, Dirven JA, et al. Application of a theoretical model to evaluate COPD disease management. BMC health services research. 2010;10(1):81.

126. Leontjevas R, Gerritsen DL, Koopmans RTCM, Smalbrugge M, Vernooij-Dassen MJFJ. Process Evaluation to Explore Internal and External Validity of the “Act in Case of Depression” Care Program in Nursing Homes. Journal of the American Medical Directors Association. 2012;13(5):488-.

127. Levac D, Glegg SMN, Sveistrup H, Colquhoun H, Miller PA, Finestone H, et al. A knowledge translation intervention to enhance clinical application of a virtual reality system in stroke rehabilitation. BMC Health Services Research. 2016;16:1-11.

128. Li F, Walker K, McInnes E, Duff J. Testing the effect of a targeted intervention on nurses’ compliance with “best practice” mechanical venous thromboembolism prevention. Journal of Vascular Nursing. 2010;28(3):92-6.

129. Liddy CE, Cullen-Arseneau P, Merizzi S, Blazhko V. "An Ounce of Prevention": A Primary Care Based Prevention Program for Pre-Diabetic Population. 2013. p. 12-7.

130. Linda M S, Gretchen P, Janice C Z. Implementing the Chronic Care Model for Improvements in Diabetes Care and Education in a Rural Primary Care Practice. The Diabetes Educator. 2005(2):225.

131. Lineker SC, Bell MJ, Boyle J, Badley EM, Flakstad L, Fleming J, et al. Implementing arthritis clinical practice guidelines in primary care. Medical Teacher. 2009;31(3):230-7.

132. Lobo CM, Euser L, Kamp J, Frijling BD, Severens JL, Hulscher ME, et al. Process evaluation of a multifaceted intervention to improve cardiovascular disease prevention in general practice. The European journal of general practice. 2003;9(3):77-83.

133. Lugtenberg M, van Beurden KM, Brouwers EPM, Terluin B, van Weeghel J, van der Klink JJL, et al. Occupational physicians' perceived barriers and suggested solutions to improve adherence to a guideline on mental health problems: analysis of a peer group training. 2016.

134. Lyndon A, Cape V. Maternal hemorrhage: Quality improvement collaborative lessons. MCN The American Journal of Maternal/Child Nursing. 2016;41(6):363-71.

135. Maas MJM, van Dulmen SA, Sagasser MH, Heerkens YF, van der Vleuten CPM, Nijhuis-van der Sanden MWG, et al. Critical features of peer assessment of clinical performance to enhance adherence to a low back pain guideline for physical therapists: a mixed methods design. 2015.

136. Martin D, Albensi L, Haute S, Froese M, Montgomery M, Lam M, et al. Healthy Skin Wins: A Glowing Pressure Ulcer Prevention Program That Can Guide Evidence-Based Practice. Worldviews on Evidence-Based Nursing. 2017;14(6):473-83.

137. McAteer J, Stone S, Fuller C, Michie S. Using psychological theory to understand the challenges facing staff delivering a ward-led intervention to increase hand hygiene behavior: A qualitative study. AJIC: American Journal of Infection Control. 2014;42(5):495-9.

138. McConnell RA, Kerlin MP, Schweickert WD, Ahmad F, Patel MS, Fuchs BD. Using a Post-Intubation Checklist and Time Out to Expedite Mechanical Ventilation Monitoring: Observational Study of a Quality Improvement Intervention. Respiratory Care. 2016;61(7):902-12.

139. McDermott L, Yardley L, Little P, van Staa T, Dregan A, McCann G, et al. Process evaluation of a point-of-care cluster randomised trial using a computer-delivered intervention to reduce antibiotic prescribing in primary care. 2014.

140. McDonnell A, Tod A, Bray K, Bainbridge D, Adsetts D, Walters S. A before and after study assessing the impact of a new model for recognizing and responding to early signs of deterioration in an acute hospital. Journal of Advanced Nursing. 2013;69(1):41-52.

141. McNamara KP, O'Reilly SL, George J, Peterson GM, Jackson SL, Duncan G, et al. Intervention Fidelity for a Complex Behaviour Change Intervention in Community Pharmacy Addressing Cardiovascular Disease Risk. Health Education Research. 2015;30(6):897-909.

142. McVey G, Gusella J, Tweed S, Ferrari M. A controlled evaluation of web-based training for teachers and public health practitioners on the prevention of eating disorders. Eating Disorders. 2008;17(1):1-26.

143. Megan C, Tina K, Larry C, Janusz K, Lisa D, Tracy G, et al. Implementing a standardized community-based cardiovascular risk assessment program in 20 Ontario communities. Health Promotion International. 2009(4):325.

144. Milne D, Woodward K, Hanner S, Iceton J, Fitzsimmons A, Rochester J. An illustration of delivering evidence-based practice through staff training: multi-dimensional process, outcome and organizational evaluation. Behavioural and Cognitive Psychotherapy. 2003;31(1):85-98.

145. Mohamad G. Fakih MM, Karen Jones RN, Janice E. Rey MT, Dorine Berriel-Cass RM, Tatyana Kalinicheva MD, Susanna Szpunar MPHD, et al. Sustained Improvements in Peripheral Venous Catheter Care in Non–Intensive Care Units: A Quasi-Experimental Controlled Study of Education and Feedback. Infection Control and Hospital Epidemiology. 2012;33(5):449.

146. Moise IK, Green D, Toth J, Mulhall PF. Evaluation of an authority innovation-decision: Brief alcohol intervention for pregnant women receiving women, infants, and children services at two illinois health departments. Substance Use and Misuse. 2014;49(7):804-12.

147. Morden A, Jinks C, Ong BN, Porcheret M, Dziedzic KS. Acceptability of a 'guidebook' for the management of Osteoarthritis: A qualitative study of patient and clinician's perspectives. BMC Musculoskeletal Disorders. 2014;15(1).

148. Mourad SM, Hermens RPMG, Liefers J, Akkermans RP, Zielhuis GA, Adang E, et al. A multi-faceted strategy to improve the use of national fertility guidelines; a cluster-randomized controlled trial. Human reproduction (Oxford Print). 2011(4):817.

149. Najavits LM, Lande RG, Gragnani C, Isenstein D, Schmitz M. Seeking safety pilot outcome study at walter reed national military medical center. Military Medicine. 2016;181(8):740-6.

150. Nancy MS, Sharon W-D, Johanne D, Janice JE, Ian DG, Susan BJ, et al. Facilitated interprofessional implementation of a physical rehabilitation guideline for stroke in inpatient settings: process evaluation of a cluster randomized trial. Implementation Science. 2017(1):1.

151. Nowalk MP, Nolan BA, Nutini J, Ahmed F, Albert SM, Susick M, et al. Success of the 4 pillars toolkit for influenza and pneumococcal vaccination in adults. Journal for Healthcare Quality. 2014;36(6):5-15.

152. Nzinga J, Ntoburi S, Wagai J, Mbindyo P, Mbaabu L, Migiro S, et al. Implementation experience during an eighteen month intervention to improve paediatric and newborn care in Kenyan district hospitals. 2009.

153. Pai M, Lloyd NS, Ji C, Thabane L, Spencer FA, Cook DJ, et al. Strategies to enhance venous thromboprophylaxis in hospitalized medical patients (SENTRY): a pilot cluster randomized trial. Implementation Science. 2013;8(1):1-11.

154. Palinkas LA, Schoenwald SK, Hoagwood K, Landsverk J, Chorpita BF, Weisz JR. An ethnographic study of implementation of evidence-based treatments in child mental health: First steps. Psychiatric services. 2008;59(7):738-46.

155. Parikh SV, Lam RW, Ovanessian MM, Filteau MJ, Hill M. Evaluation of a disease‐management intervention designed to reduce depression disability. Journal of evaluation in clinical practice. 2011;17(2):322-5.

156. Parsons JA, Yu CHY, Baker NA, Mamdani MM, Bhattacharyya O, Zwarenstein M, et al. Practice Doesn’t Always Make Perfect: A Qualitative Study Explaining Why a Trial of an Educational Toolkit Did Not Improve Quality of Care. PLOS ONE. 2016;11(12):e0167878.

157. Patricia H, Valerie S, Gerard S, Mike C, Declan D, Mechthild MG, et al. Process evaluation for OptiBIRTH, a randomised controlled trial of a complex intervention designed to increase rates of vaginal birth after caesarean section. Trials. 2018(1):1.

158. Patricia H, William H, Carmel M, Enrique S, Adriana N. A Process Evaluation of an Intervention to Improve Respiratory Infection Control Practices in Family Physician Offices. Canadian Journal of Public Health / Revue Canadienne de Sante'e Publique. 2006;97(6):475.

159. Penny B, David R, Samantha L, John B, Lorna B, Karina L. Specialist educational intervention for mental health nursing staff: delivery, content and personal impact. Journal of Advanced Nursing. 2005(5):529.

160. Pereles L, Lockyer J, Ryan D, Davis D, Spivak B, Robinson B. The use of the opinion leader in continuing medical education. Medical Teacher. 2003;25(4):438-41.

161. Pérez-Granda MJ, Rincón C, Guembe M, Muñoz P, Bouza E. Effectiveness of a training program in compliance with recommendations for venous lines care. BMC Infectious Diseases. 2015;15(1).

162. PLASS AMC, LEMS WF, VOSKUYL AE, KERSTENS PJ, DIJKMANS BA, BOERS M. Facilitating the use of COBRA combination therapy in early rheumatoid arthritis: a pilot implementation study. The Journal of rheumatology. 2009;36(7):1380-6.

163. Ploeg J, Davies B, Edwards N, Gifford W, Miller PE. Factors influencing best‐practice guideline implementation: Lessons learned from administrators, nursing staff, and project leaders. Worldviews on Evidence‐Based Nursing. 2007;4(4):210-9.

164. Possemato K, Johnson EM, Webster B, Wray LO, Stecker T. The implementation and testing of a referral management system to address barriers to treatment seeking among primary care veterans with PTSD. Psychological Services. 2018;15(4):457-69.

165. Presseau J, Grimshaw JM, Tetroe JM, Eccles MP, Francis JJ, Godin G, et al. A theory-based process evaluation alongside a randomised controlled trial of printed educational messages to increase primary care physicians' prescription of thiazide diuretics for hypertension [ISRCTN72772651]. 2016.

166. Prieto J, Clark JM. Contact precautions for Clostridium dif.cile and Methicillin-resistant Staphylococcus aureus (MRSA):Assessing the impact of a supportive intervention to improve practice. Journal of Research in Nursing. 2005;10(5):511-26.

167. Purvis T, Moss K, Denisenko S, Bladin C, Cadilhac DA. Implementation of evidence-based stroke care: enablers, barriers, and the role of facilitators. Journal of Multidisciplinary Healthcare. 2014(default):389.

168. Rachel G, Arwen B, Stuart C, James VD, Celine H, Christine AN, et al. Cardiovascular care guideline implementation in community health centers in Oregon: a mixed-methods analysis of real-world barriers and challenges. BMC Health Services Research. 2017(1):1.

169. Ragazzi H, Keller A, Ehrensberger R, Irani A-M. Evaluation of a practice-based intervention to improve the management of pediatric asthma. Journal of Urban Health. 2011;88(1):38-48.

170. Rajasekhar PT, Rees CJ, Nixon C, East JE, Brown S. Factors influencing change in clinical practice: A qualitative evaluation of the implementation of the quality improvement in colonoscopy study. International Journal of Health Care Quality Assurance. 2016;29(1):5-15.

171. Ramsay CR, Thomas RE, Croal BL, Grimshaw JM, Eccles MP. Using the theory of planned behaviour as a process evaluation tool in randomised trials of knowledge translation strategies: A case study from UK primary care. 2010.

172. Redfern S, Christian S. Achieving change in health care practice. Journal of Evaluation in Clinical Practice. 2003;9(2):225-38.

173. Renata TM, Anna LB, Darshini RA, Fiona L, Jeannette K, Keith DH, et al. Implementation fidelity of a nurse-led falls prevention program in acute hospitals during the 6-PACK trial. BMC Health Services Research. 2017(1):1.

174. Reper P, Dicker D, Damas P, Huyghens L, Haelterman M. Improving the quality of the intensive care follow-up of ventilated patients during a national registration program. Public Health. 2017;148:159-66.

175. Resnick B, Quinn C, Baxter S. Testing the Feasibility of Implementation of Clinical Practice Guidelines in Long-Term Care Facilities. Journal of the American Medical Directors Association. 2004;5(1):1-8.

176. Richmond H, Hall AM, Hansen Z, Williamson E, Davies D, Lamb SE. Using mixed methods evaluation to assess the feasibility of online clinical training in evidence based interventions: a case study of cognitive behavioural treatment for low back pain. 2016.

177. Richter-Sundberg L, Nystrom ME, Krakau I, Sandahl C. Improving treatment of depression in primary health care: a case study of obstacles to perform a clinical trial designed to implement practice guidelines. 2015. p. 188-200.

178. Ricketts EJ, Francischetto EOC, Wallace LM, Hogan A, McNulty CAM. Tools to overcome potential barriers to chlamydia screening in general practice: Qualitative evaluation of the implementation of a complex intervention. 2016.

179. Roberts S, McInnes E, Bucknall T, Wallis M, Banks M, Chaboyer W. Process evaluation of a cluster-randomised trial testing a pressure ulcer prevention care bundle: a mixed-methods study. 2017.

180. Rycroft-Malone J, Hawkes CA, Seers K, Chandler J, Allen C, Crichton N, et al. The role of evidence, context, and facilitation in an implementation trial: Implications for the development of the PARIHS framework. Implementation Science. 2013;8(1).

181. Rycroft-Malone J, Seers K, Crichton N, Chandler J, Hawkes CA, Allen C, et al. A pragmatic cluster randomised trial evaluating three implementation interventions. Implementation Science. 2012;7(1):80.

182. S S, A C, A B, G V, F M, S F, et al. Improving healthcare worker hand hygiene adherence before patient contact: a before-and-after five-unit multimodal intervention in Tuscany. Quality & Safety in Health Care. 2009(6):429.

183. Sachdeva R, Kelleman MS, McCracken CE, Campbell RM, Lai WW, Lopez L, et al. Physician Attitudes toward the First Pediatric Appropriate Use Criteria and Engagement With Educational Intervention to Improve the Appropriateness of Outpatient Echocardiography. Journal of the American Society of Echocardiography. 2017;30(9):926-31.

184. Sanchez A, Grandes G, Cortada JM, Pombo H, Martinez C, Corrales MH, et al. Feasibility of an implementation strategy for the integration of health promotion in routine primary care: a quantitative process evaluation. 2017.

185. Sanchez A, Silvestre C, Campo N, Grandes G, Pre DERG. Type-2 diabetes primary prevention program implemented in routine primary care: a process evaluation study. 2016.

186. Sanders S, Mackin ML, Reyes J, Herr K, Titler M, Fine P, et al. Implementing Evidence-Based Practices: Considerations for the Hospice Setting. 2010. p. 369-76.

187. Sanders T, Nio Ong B, Sowden G, Foster N. Implementing change in physiotherapy: professions, contexts and interventions. Journal of Health Organization and Management. 2014(1):96.

188. Sarah EPM, Ian DG, Nancy MS, Susan BJ, Carol LR, Janice JE, et al. Perspectives of health care professionals on the facilitators and barriers to the implementation of a stroke rehabilitation guidelines cluster randomized controlled trial. BMC Health Services Research. 2017(1):1.

189. Sevón T, Heikkilä K, Hemminki E, Koponen P. Special features of health services and register based trials – experiences from a randomized trial of childbirth classes. BMC Health Services Research. 2008(1):126.

190. Sharma M, Deepak S. Rehabilitation in practice. A participatory evaluation of community-based rehabilitation programme in North Central Vietnam. Disability & Rehabilitation. 2001;23(8):352-8.

191. Sheth H, Moreland L, Peterson H, Aggarwal R. Improvement in Herpes Zoster Vaccination in Patients with Rheumatoid Arthritis: A Quality Improvement Project. 2017.

192. Sibyl A, Veronique V, Olivier S, Samuel C. Academic detailers’ and general practitioners’ views and experiences of their academic detailing visits to improve the quality of analgesic use: process evaluation alongside a pragmatic cluster randomized controlled trial. BMC Health Services Research. 2017(1):1.

193. Siddiqi N, Young J, House AO, Featherstone I, Hopton A, Martin C, et al. Stop Delirium! A complex intervention to prevent delirium in care homes: a mixed-methods feasibility study. 2011. p. 90-8.

194. Simpson MR, Stevens P, Kovach CR. Nurses' experience with the clinical application of a research-based nursing protocol in a long-term care setting. Journal of Clinical Nursing (Wiley-Blackwell). 2007;16(6):1021-8.

195. Simunovic M, Stephen W, Kelly S, Forbes S, Cadeddu M, Thabane L, et al. Quality Improvement in Colorectal Cancer in Local Health Integration Network 4 (LHIN 4) Project (QICC-L4): Integrated Knowledge Translation in a Large Geographic Region. 2013. p. 4067-72.

196. Singh M, Hynie M, Rivera T, MacIsaac L, Gladman A, Cheng A. An evaluation study of the implementation of stroke best practice guidelines using a Knowledge Transfer Team approach. Canadian Journal of Neuroscience Nursing. 2015;37(1):24-36.

197. Sinnema H, Volker D, Franx G, De Lange J, Terluin B, Wensing M, et al. Systematic tailoring for the implementation of guideline recommendations for anxiety and depressive disorders in general practice: Perceived usefulness of tailored interventions. BMC Family Practice. 2013;14.

198. Sodhi S, Banda H, Kathyola D, Joshua M, Richardson F, Mah E, et al. Supporting middle-cadre health care workers in Malawi: lessons learned during implementation of the PALM PLUS package. 2014.

199. Solomon JL, Bokhour BG, Butler J, Golden JF, Hare K, Kertz B, et al. Sustaining Nurse‐Rapid HIV Testing in the US Department of Veterans Affairs: Lessons Learned from a Comparative Evaluation. Journal for Healthcare Quality. 2014;36(5):26-31.

200. Sopcak N, Aguilar C, Manca DP, O'Brien MA, Grunfeld E, Nykiforuk C, et al. Implementation of the BETTER 2 program: A qualitative study exploring barriers and facilitators of a novel way to improve chronic disease prevention and screening in primary care. Implementation Science. 2016;11(1).

201. Stafseth SK, Grønbeck S, Lien T, Randen I, Lerdal A. The experiences of nurses implementing the Modified Early Warning Score and a 24-hour on-call Mobile Intensive Care Nurse: An exploratory study. Intensive and Critical Care Nursing. 2016;34:33-41.

202. Stein J, Lewin S, Fairall L, Mayers P, English R, Bheekie A, et al. Building capacity for antiretroviral delivery in South Africa: a qualitative evaluation of the PALSA PLUS nurse training programme. BMC Health Services Research. 2008;8(1):240.

203. Steinmo SH, Michie S, Fuller C, Stanley S, Stapleton C, Stone SP. Bridging the gap between pragmatic intervention design and theory: using behavioural science tools to modify an existing quality improvement programme to implement "Sepsis Six". 2016.

204. Stevens BJ, Yamada J, Promislow S, Stinson J, Harrison D, Victor JC, et al. Implementation of multidimensional knowledge translation strategies to improve procedural pain in hospitalized children. 2014.

205. Stone S, Prater L, Spencer R. Facilitating Skin‐to‐Skin Contact in the Operating Room After Cesarean Birth. Nursing for Women’s Health. 2014;18(6):486-99.

206. Sturkenboom IHWM, Nijhuis-van der Sanden MWG, Graff MJL. A process evaluation of a home-based occupational therapy intervention for Parkinson’s patients and their caregivers performed alongside a randomized controlled trial. Clinical Rehabilitation. 2016;30(12):1186-99.

207. Sullivan DJ, Antle BF, van Zyl MA, Faul AC. Integrating Evidence-Based Practice into Public Mental Health Settings: revaluation of the Kentucky Medication Algorithm Program. Best Practice in Mental Health. 2009;5(2):112-28.

208. Sunaert P, Bastiaens H, Feyen L, Snauwaert B, Nobels F, Wens J, et al. Implementation of a program for type 2 diabetes based on the Chronic Care Model in a hospital-centered health care system:" the Belgian experience". BMC Health Services Research. 2009;9(1):152.

209. Thomas LH, French B, Burton CR, Sutton C, Forshaw D, Dickinson H, et al. Evaluating a systematic voiding programme for patients with urinary incontinence after stroke in secondary care using soft systems analysis and Normalisation Process Theory: Findings from the ICONS case study phase. International Journal of Nursing Studies. 2014;51(10):1308-20.

210. Tierney S, Kislov R, Deaton C. A qualitative study of a primary-care based intervention to improve the management of patients with heart failure: The dynamic relationship between facilitation and context. BMC Family Practice. 2014;15(1).

211. Tony F, Elizabeth B, Michelle C, Desley H, Debra M, Judith M, et al. Implementation of oral health recommendations into two residential aged care facilities in a regional Australian city. International Journal of Evidence-Based Healthcare. 2006(3):162.

212. Toth-Pal E, Wårdh I, Strender L-E, Nilsson G. Implementing a clinical decision-support system in practice: A qualitative analysis of influencing attitudes and characteristics among general practitioners. Informatics for Health & Social Care. 2008;33(1):39-54.

213. Valanis B, Labuhn KT, Stevens NH, Lichtenstein E, Brody KK. Integrating prenatal-postnatal smoking interventions into usual care in a health maintenance organization. Health promotion practice. 2003;4(3):236-48.

214. Van den Branden S, Van den Broucke S, Leroy R, Declerck D, Hoppenbrouwers K. Evaluating the Implementation Fidelity of a Multicomponent Intervention for Oral Health Promotion in Preschool Children. 2015. p. 1-10.

215. Van Der Meer EWC, Boot CRL, Jungbauer FHW, Coenraads PJ, Van Der Gulden JWJ, Anema JR. Implementation of Recommendations for Hand Eczema Through a Multifaceted Strategy. A Process Evaluation Among Health Care Workers. Acta Dermato-Venereologica. 2014;94(6):651-7.

216. van Os-Medendorp H, Kok PE-d, van Linge R, Bruijnzeel-Koomen C, Grypdonck M, Ros W. The tailored implementation of the nursing programme 'Coping with itch'. 2008. p. 1460-70.

217. van Weert JCM, Kerkstra A, van Dulmen AM, Bensing JM, Peter JG, Ribbe MW. The implementation of snoezelen in psychogeriatric care: an evaluation through the eyes of caregivers. International Journal of Nursing Studies. 2004;41(4):397-409.

218. Varghese B, Krishnamurthy J, Panigrahi R, Correia B, Washington M, Ponnuswamy V, et al. Limited effectiveness of a skills and drills intervention to improve emergency obstetric and newborn care in Karnataka, India: A proof-of-concept study. Global Health Science and Practice. 2016;4(4):582-93.

219. Vos AA, van Voorst SF, Posthumus AG, Waelput AJM, Denktaş S, Steegers EAP. Process evaluation of the implementation of scorecard-based antenatal risk assessment, care pathways and interdisciplinary consultation: the Healthy Pregnancy 4 All study. Public Health. 2017;150:112-20.

220. Wang Y, Xiao LD, Ullah S, He G-P, De Bellis A. Evaluation of a nurse-led dementia education and knowledge translation programme in primary care: A cluster randomized controlled trial. Nurse Education Today. 2017;49:1-7.

221. Weili LU, Yanos PT, Gottlieb JD, Marcello Duva S, Silverstein SM, Haiyi XIE, et al. Use of Fidelity Assessments to Train Clinicians in the CBT for PTSD Program for Clients With Serious Mental Illness : Gary Bond and Fidelity Assessment. Psychiatric services (Washington, DC). 2012(8):785.

222. Whitley R, Gingerich S, Lutz WJ, Mueser KT. Implementing the Illness Management and Recovery Program in Community Mental Health Settings : Facilitators and Barriers. Psychiatric services (Washington, DC). 2009(2):202.

223. Wieczorek B, Ascenzi J, Kim Y, Lenker H, Potter C, Shata NJ, et al. PICU Up!: Impact of a Quality Improvement Intervention to Promote Early Mobilization in Critically Ill Children. 2016. p. E559-E66.

224. Wilson DS, Montie M, Titler MG, Conlon P, Reynolds M, Ripley R. Nurses’ Perceptions of Implementing Fall Prevention Interventions to Mitigate Patient-Specific Fall Risk Factors. Western Journal of Nursing Research. 2016;38(8):1012-34.

225. Wright IM, Wake CH, Anderson H, Graham S. Assessment of the multidisciplinary education for a major change in clinical practice; a prospective cohort study. BMC health services research. 2009;9(1):28.

226. Zapka J, Goins KV, Pbert L, Ockene JK. Translating Efficacy Research to Effectiveness Studies in Practice: Lessons From Research to Promote Smoking Cessation in Community Health Centers. Health Promotion Practice. 2004;5(3):245-55.
