# Supplementary material for: Systematic review of the use of process evaluations in knowledge translation research
Source: Syst Rev. 2019 Nov 7;8:266. doi: 10.1186/s13643-019-1161-y (PMC6836407; doi:10.1186/s13643-019-1161-y)
Supplement: Supplementary file 1 — Additional file 1. Peer-reviewed Search Strategy. Systematic literature review search documenation. [file 13643_2019_1161_MOESM1_ESM.docx]

**Additional File 1:** Peer-Reviewed Search Strategy

**Ovid MEDLINE(R) In-Process & Other Non-Indexed Citations**

**Search date:** May 24, 2018

***Search terms/criteria:***

1. evidence-based practice/ or evidence-based dentistry/ or evidence-based medicine/ or evidence-based emergency medicine/ or evidence-based nursing/

2. guideline adherence/ or quality assurance, health care/ or guidelines as topic/ or practice guidelines as topic/ or quality improvement/

3. "diffusion of innovation"/ or Translational Medical Research/

4. (evidence based or evidence into practice).ti.

5. (evidence based or evidence into practice).ab. /freq=2

6. ((cpg* or guideline*) adj2 (implement* or adher* or adoption)).ti.

7. (quality improv* adj3 (health care or healthcare)).tw.

8. (research adj2 ("use" or utili?ation or utili?e or adopt* or implement* or disseminat* or uptake)).tw.

9. (research findings adj2 (transfer* or translat*)).tw.

10. (knowledge adj2 ("use" or utili?ation or utili?e or adopt* or implement* or disseminat* or uptake or transfer* or translat*)).tw.

11. (evidence adj2 (utili?ation or utili?e or adopt* or implement* or disseminat* or uptake or transfer* or translat*)).tw.

12. (innovation adj2 adopt*).tw.

13. ((organi?ational or complex) adj2 intervention).tw.

14. (implementation adj3 research).tw.

15. implementation science.tw.

16. ((change or improv*) adj2 practice).tw.

17. or/1-16

18. Health Plan Implementation/ or Capacity Building/

19. (implement* or intervention* or program* or project or intitiative).ti.

20. (implement* or intervention* or program* or project or intitiative).ab. /freq=3

21. or/18-20

22. 17 and 21

23. "process assessment (health care)"/ or program evaluation/ or qualitative research/

24. (black box or process evaluation* or qualitative component* or qualitative aspect* or qualitative approach* or systematic evaluation* or participant observation or simulation or implementation audit).mp.

25. (audit or feedback).tw.

26. or/23-25

27. 22 and 26

28. ("8607003" or "8805847" or "101088677" or "8303205" or "9609066" or "101247063" or "101170481" or "101258411").jc.

29. 28 and 26

30. 27 or 29

31. limit 30 to (english language and yr="1996 -Current")

**Ovid Medline 1946-**

**Search date:** May 24, 2018

***Search terms/criteria:***

1. evidence-based practice/ or evidence-based dentistry/ or evidence-based medicine/ or evidence-based emergency medicine/ or evidence-based nursing/

2. guideline adherence/ or quality assurance, health care/ or guidelines as topic/ or practice guidelines as topic/ or quality improvement/

3. "diffusion of innovation"/ or Translational Medical Research/

4. (evidence based or evidence into practice).ti.

5. (evidence based or evidence into practice).ab. /freq=2

6. ((cpg* or guideline*) adj2 (implement* or adher* or adoption)).ti.

7. (quality improv* adj3 (health care or healthcare)).tw.

8. (research adj2 ("use" or utili?ation or utili?e or adopt* or implement* or disseminat* or uptake)).tw.

9. (research findings adj2 (transfer* or translat*)).tw.

10. (knowledge adj2 ("use" or utili?ation or utili?e or adopt* or implement* or disseminat* or uptake or transfer* or translat*)).tw.

11. (evidence adj2 (utili?ation or utili?e or adopt* or implement* or disseminat* or uptake or transfer* or translat*)).tw.

12. (innovation adj2 adopt*).tw.

13. ((organi?ational or complex) adj2 intervention).tw.

14. (implementation adj3 research).tw.

15. implementation science.tw.

16. ((change or improv*) adj2 practice).tw.

17. or/1-16

18. Health Plan Implementation/ or Capacity Building/

19. (implement* or intervention* or program* or project or intitiative).ti.

20. (implement* or intervention* or program* or project or intitiative).ab. /freq=3

21. or/18-20

22. 17 and 21

23. "process assessment (health care)"/ or program evaluation/ or qualitative research/

24. (black box or process evaluation* or qualitative component* or qualitative aspect* or qualitative approach* or systematic evaluation* or participant observation or simulation or implementation audit).mp.

25. (audit or feedback).tw.

26. or/23-25

27. 22 and 26

28. ("8607003" or "8805847" or "101088677" or "8303205" or "9609066" or "101247063" or "101170481" or "101258411").jc.

29. 28 and 26

30. 27 or 29

31. limit 30 to (english language and yr="1996 -Current")

**Ovid Embase 1996-**

**Search date:** May 24, 2018

***Search terms/criteria:***

1. *evidence based practice/ or *evidence based dentistry/ or *evidence based medicine/ or *evidence based nursing/

2. *practice guideline/ or *total quality management/

3. mass communication/ or *translational research/

4. (evidence based or evidence into practice).ti.

5. (evidence based or evidence into practice).ab. /freq=2

6. ((cpg* or guideline*) adj2 (implement* or adher* or adoption)).ti.

7. (quality improv* adj3 (health care or healthcare)).tw.

8. (research adj2 ("use" or utili?ation or utili?e or adopt* or implement* or disseminat* or uptake)).tw.

9. (research findings adj2 (transfer* or translat*)).tw.

10. (knowledge adj2 ("use" or utili?ation or utili?e or adopt* or implement* or disseminat* or uptake or transfer* or translat*)).tw.

11. (evidence adj2 (utili?ation or utili?e or adopt* or implement* or disseminat* or uptake or transfer* or translat*)).tw.

12. (innovation adj2 adopt*).tw.

13. ((organi?ational or complex) adj2 intervention).tw.

14. (implementation adj3 research).tw.

15. implementation science.tw.

16. ((change or improv*) adj2 practice).tw.

17. or/1-16

18. *Capacity Building/

19. (implement* or intervention* or program* or project or intitiative).ti.

20. (implement* or intervention* or program* or project or intitiative).ab. /freq=3

21. or/18-20

22. 17 and 21

23. *evaluation/ or qualitative research/

24. (black box or process evaluation* or qualitative component* or qualitative aspect* or qualitative approach* or systematic evaluation* or participant observation or simulation or implementation audit).mp.

25. (audit or feedback).tw.

26. or/23-25

27. 22 and 26

28. limit 27 to (english language and yr="1996 -Current")

**Ovid PsycINFO 1987-**

**Search date:** May 24, 2018

***Search terms/criteria:***

1. evidence based practice/

2. treatment guidelines/

3. innovation/ or knowledge transfer/

4. (evidence based or evidence into practice).ti.

5. (evidence based or evidence into practice).ab. /freq=2

6. ((cpg* or guideline*) adj2 (implement* or adher* or adoption)).ti.

7. (quality improv* adj3 (health care or healthcare)).tw.

8. (research adj2 ("use" or utili?ation or utili?e or adopt* or implement* or disseminat* or uptake)).tw.

9. (research findings adj2 (transfer* or translat*)).tw.

10. (knowledge adj2 ("use" or utili?ation or utili?e or adopt* or implement* or disseminat* or uptake or transfer* or translat*)).tw.

11. (evidence adj2 (utili?ation or utili?e or adopt* or implement* or disseminat* or uptake or transfer* or translat*)).tw.

12. (innovation adj2 adopt*).tw.

13. ((organi?ational or complex) adj2 intervention).tw.

14. (implementation adj3 research).tw.

15. implementation science.tw.

16. ((change or improv*) adj2 practice).tw.

17. or/1-16

18. (implement* or intervention* or program* or project or intitiative).ti.

19. (implement* or intervention* or program* or project or intitiative).ab. /freq=3

20. 18 or 19

21. 17 and 20

22. program evaluation/ or evaluation/ or educational program evaluation/ or qualitative research/ or grounded theory/ or exp interviews/ or observation methods/

23. (black box or process evaluation* or qualitative component* or qualitative aspect* or qualitative approach* or systematic evaluation* or participant observation or simulation or implementation audit).mp.

24. (audit or feedback).tw.

25. or/22-24

26. 21 and 25

27. limit 26 to (english language and yr="1996 -Current")

**EBSCOhost CINAHL 1937-**

**Search date:** May 24, 2018

***Search terms/criteria:***

| **Search Terms** | **Search Options** | **Actions** |
| --- | --- | --- |
| S31 | S23 AND S28 | **Limiters** - English Language; Published Date: 19960101-; Research Article  **Search modes** - Find all my search terms |
| S30 | S23 AND S28 | **Limiters** - Published Date: 19960101-; Research Article  **Search modes** - Find all my search terms |
| S29 | S23 AND S28 | **Search modes** - Find all my search terms |
| S28 | S24 OR S25 OR S26 OR S27 | **Search modes** - Find all my search terms |
| S27 | audit or feedback | **Search modes** - Find all my search terms |
| S26 | "black box" or "process evaluation*" or "qualitative component*" or "qualitative aspect*" or "qualitative approach*" or "systematic evaluation*" or "participant observation" or simulation or "implementation audit" | **Search modes** - Find all my search terms |
| S25 | (MH "Qualitative Studies+") | **Search modes** - Find all my search terms |
| S24 | (MH "Process Assessment (Health Care)+") OR (MH "Program Evaluation") | **Search modes** - Find all my search terms |
| S23 | S19 AND S22 | **Search modes** - Find all my search terms |
| S22 | S20 OR S21 | **Search modes** - Find all my search terms |
| S21 | TI implement* or intervention* or program* or project or intitiative | **Search modes** - Find all my search terms |
| S20 | (MH "Program Implementation") | **Search modes** - Find all my search terms |
| S19 | S1 OR S2 OR S3 OR S4 OR S5 OR S6 OR S7 OR S8 OR S9 OR S10 OR S11 OR S12 OR S13 OR S14 OR S15 OR S16 OR S17 OR S18 | **Search modes** - Find all my search terms |
| S18 | change N2 practice OR improv* N2 practice | **Search modes** - Find all my search terms |
| S17 | change N2 practice OR improve N2 practice | **Search modes** - Find all my search terms |
| S16 | "implementation science" | **Search modes** - Find all my search terms |
| S15 | implementation N2 research | **Search modes** - Find all my search terms |
| S14 | complex N2 intervention | **Search modes** - Find all my search terms |
| S13 | organi?ational N2 intervention | **Search modes** - Find all my search terms |
| S12 | innovation N2 adopt* | **Search modes** - Find all my search terms |
| S11 | evidence N2 (utili?ation or adopt* or implement* or disseminat* or uptake or transfer* or translat*) | **Search modes** - Find all my search terms |
| S10 | knowledge N2 ("use" or utili?ation or adopt* or implement* or disseminat* or uptake or transfer* or translat*) | **Search modes** - Find all my search terms |
| S9 | "research findings" N2 transfer* OR "research findings" N2 translat* | **Search modes** - Find all my search terms |
| S8 | research N2 ("use" or utili?ation or adopt* or implement* or disseminat* or uptake) | **Search modes** - Find all my search terms |
| S7 | "quality improv*" N3 "health care" OR "quality improv*" N3 healthcare | **Search modes** - Find all my search terms |
| S6 | TI guideline* N2 implement* OR TI guideline* N2 adher* OR TI guideline* N2 adoption | **Search modes** - Find all my search terms |
| S5 | TI evidence based or evidence into practice | **Search modes** - Find all my search terms |
| S4 | (MH "Diffusion of Innovation") | **Search modes** - Find all my search terms |
| S3 | (MH "Quality Improvement+") OR (MH "Quality Assurance") | **Search modes** - Find all my search terms |
| S2 | (MH "Guideline Adherence") | **Search modes** - Find all my search terms |
| S1 | (MH "Professional Practice, Evidence-Based+") | **Search modes** - Find all my search terms |

**Web of Science**

**Search date:** May 24, 2018

***Search terms/criteria:***

Science Citation Index Expanded (SCI-EXPANDED) --1900-present

Social Sciences Citation Index (SSCI) --1900-present

Conference Proceedings Citation Index- Science (CPCI-S) --1990-present

Conference Proceedings Citation Index- Social Science & Humanities (CPCI-SSH) --1990-present

| #21 | (#20 OR #19) AND Language=(English)  *Databases=SCI-EXPANDED, SSCI, CPCI-S, CPCI-SSH Timespan=1996-2018* |
| --- | --- |
| #20 | #17 AND #16  *Databases=SCI-EXPANDED, SSCI, CPCI-S, CPCI-SSH Timespan=1996-2018* |
| #19 | #18 AND #16  *Databases=SCI-EXPANDED, SSCI, CPCI-S, CPCI-SSH Timespan=1996-2018* |
| #18 | TS=("qualitative research")  *Databases=SCI-EXPANDED, SSCI, CPCI-S, CPCI-SSH Timespan=1996-2018* |
| #17 | TS=("black box" or "process evaluation*" or "qualitative component*" or "qualitative aspect*" or "qualitative approach*" or "systematic evaluation*" or "participant observation" or simulation or "implementation audit")  *Databases=SCI-EXPANDED, SSCI, CPCI-S, CPCI-SSH Timespan=1996-2018* |
| #16 | #15 AND #14  *Databases=SCI-EXPANDED, SSCI, CPCI-S, CPCI-SSH Timespan=1996-2018* |
| #15 | TI=(implement* or intervention* or program* or project or intitiative)  *Databases=SCI-EXPANDED, SSCI, CPCI-S, CPCI-SSH Timespan=1996-2018* |
| #14 | #13 OR #12 OR #11 OR #10 OR #9 OR #8 OR #7 OR #6 OR #5 OR #4 OR #3 OR #2 OR #1  *Databases=SCI-EXPANDED, SSCI, CPCI-S, CPCI-SSH Timespan=1996-2018* |
| #13 | TS=(change NEAR/2 practice) OR TS=(improv* NEAR/2 practice)  *Databases=SCI-EXPANDED, SSCI, CPCI-S, CPCI-SSH Timespan=1996-2018* |
| #12 | TS=("implementation science")  *Databases=SCI-EXPANDED, SSCI, CPCI-S, CPCI-SSH Timespan=1996-2018* |
| #11 | TS=(implementation NEAR/3 research)  *Databases=SCI-EXPANDED, SSCI, CPCI-S, CPCI-SSH Timespan=1996-2018* |
| #10 | TS=(organi?ational NEAR/2 intervention) OR TS=(complex NEAR/2 intervention)  *Databases=SCI-EXPANDED, SSCI, CPCI-S, CPCI-SSH Timespan=1996-2018* |
| #9 | TS=(innovation NEAR/2 adopt*)  *Databases=SCI-EXPANDED, SSCI, CPCI-S, CPCI-SSH Timespan=1996-2018* |
| #8 | TS=(evidence NEAR/2 utili?ation) OR TS=(evidence NEAR/2 utili?e) OR TS=(evidence NEAR/2 adopt*) OR TS=(evidence NEAR/2 implement*) OR TS=(evidence NEAR/2 disseminat*) OR TS=(evidence NEAR/2 uptake) OR TS=(evidence NEAR/2 transfer) OR TS=(evidence NEAR/2 translation)  *Databases=SCI-EXPANDED, SSCI, CPCI-S, CPCI-SSH Timespan=1996-2018* |
| #7 | TS=("research findings" NEAR/2 transfer) OR TS=("research findings" NEAR/2 translation)  *Databases=SCI-EXPANDED, SSCI, CPCI-S, CPCI-SSH Timespan=1996-2018* |
| #6 | TS=(knowledge NEAR/2 "use") OR TS=(knowledge NEAR/2 utili?ation) OR TS=(knowledge NEAR/2 utili?e) OR TS=(knowledge NEAR/2 adopt*) OR TS=(knowledge NEAR/2 implement*) OR TS=(knowledge NEAR/2 disseminat*) OR TS=(knowledge NEAR/2 uptake) OR TS=(knowledge NEAR/2 transfer) OR TS=(knowledge NEAR/2 translation)  *Databases=SCI-EXPANDED, SSCI, CPCI-S, CPCI-SSH Timespan=1996-2018* |
| #5 | TS=(research NEAR/2 "use") OR TS=(research NEAR/2 utili?ation) OR TS=(research NEAR/2 utili?e) OR TS=(research NEAR/2 adopt*) OR TS=(research NEAR/2 implement*) OR TS=(research NEAR/2 disseminat*) OR TS=(research NEAR/2 uptake)  *Databases=SCI-EXPANDED, SSCI, CPCI-S, CPCI-SSH Timespan=1996-2018* |
| #4 | TS=("quality improv*" NEAR/3 "health care") OR TS=("quality improv*" NEAR/3 healthcare)  *Databases=SCI-EXPANDED, SSCI, CPCI-S, CPCI-SSH Timespan=1996-2018* |
| #3 | TS=("diffusion of innovation")  *Databases=SCI-EXPANDED, SSCI, CPCI-S, CPCI-SSH Timespan=1996-2018* |
| #2 | TI=(guideline* NEAR/2 implement*) OR TI=(guideline* NEAR/2 adher*) OR TI=(guideline* NEAR/2 adoption)  *Databases=SCI-EXPANDED, SSCI, CPCI-S, CPCI-SSH Timespan=1996-2018* |
| #1 | TI=("evidence based" or "evidence into practice")  Databases=SCI-EXPANDED, SSCI, CPCI-S, CPCI-SSH Timespan=1996-2018 |

**ProQuest ProQuest Dissertations & Theses Full Text**

**Search date:** May 24, 2018

(((ti("evidence based" OR "evidence into practice") OR ti("guideline* implement*" OR "guideline* adher*" OR "guideline adoption") OR all("diffusion of innovation") OR (all("quality improvement") AND all((healthcare OR "health care"))) OR all("research use" OR "research utili?ation" OR "utili?e research " OR "research adopt*" OR "research implement*" OR "research disseminat*" OR "research uptake") OR all("knowledge use" OR "knowledge utilisation" OR "knowledge utilisation" OR "utilize knowledge" OR "utilise knowledge" OR "knowledge adopt*" OR "knowledge implementation" OR "knowledge disseminat*" OR "knowledge uptake" OR "knowledge transfer" OR "knowledge translation") OR all("evidence utilisation" OR "evidence utilization" OR "utilize evidence" OR "utilise evidence" OR "evidence adopt*" OR "evidence implement*" OR "evidence disseminat*" OR "evidence uptake" OR "evidence transfer" OR "evidence translation") OR all(innovation adoption OR complex intervention OR implementation research OR implementation science)) AND ti(implement* or intervention* or program* or project or intitiative)) AND (all("black box" OR "process evaluation*" OR "qualitative component*" OR "qualitative aspect*" OR "qualitative approach*" OR "systematic evaluation*" OR "participant observation" OR simulation OR "implementation audit") OR all("qualitative research"))) AND all(health OR healthcare OR "health care" OR nurs* OR medic* OR therap* OR physician* OR hospital* OR clinic*)

Publication date: 1996 - 2018
